# The Sulfonation of Acetone: Characterization of Acetonedisulfonic Acid and Its Salts

**DOI:** 10.1002/cplu.70237

**Published:** 2026-09-07

**Authors:** Jan Langwald, Jan Chrubasik, Antonio Frontera, Mathias S. Wickleder

**Affiliations:** ^1^ Institute of Inorganic and Materials Chemistry University of Cologne Cologne Germany; ^2^ Department of Chemistry Institute of Organic Chemistry University of the Balearic Islands Palma de Mallorca Spain

**Keywords:** hydrogen bonds, noncovalent interactions organosulfonates, organosulfonates, solid state structures, sulfonation

## Abstract

We report the synthesis of acetonedisulfonic acid ([(HOSO_2_CH_2_)_2_CO], ADSA) by slow reaction of acetone and oleum (65% SO_3_) and subsequent removal of H_2_O. The new acid was used to prepare potassium acetonedisulfonate, K_2_[ADS] (ADS = [(OSO_2_CH_2_)_2_CO]^2–^), as well as the rubidium and the pyridinium salts, Rb_2_[ADS] and [PyH]_3_[ADS][HSO_4_], respectively. Furthermore, we present an alternative one‐pot reaction yielding the ammonium salt [NH_4_]_2_[ADS], which we characterized spectroscopically. The latter was used for a subsequent salt metathesis reaction for the preparation of the strontium and calcium salts. For the first time, all these compounds were structurally elucidated by single‐crystal X‐ray diffraction methods (SCXRD), revealing different conformers that the [ADS]^2–^ anion may adopt. Deeper insights into the structural versatility of the new compounds were gained by quantum chemical calculations. These investigations led to the molecular electrostatic potential (MEP) surfaces of the acid, as well as of the [NH_4_]^+^ and the [C_5_H_6_N]^+^ salt, mapping the energetic landscape of the different species as a tool to predict the different reactivities. Subsequently, energetic and *Bader’s* quantum theory of atoms in molecules (QTAIM) analyses were used to show the stabilization potential of the strong hydrogen bonds present in all of the three species and their influence on the intramolecular assemblies found in the solid state.

## Graphical abstract

Acetonedisulfonic acid and some of its salts were structurally elucidated. Depending on the counterion, either strong hydrogen bonding or different coordination behaviors are found, showing the potential tunability of functionalized (organo)sulfonates.

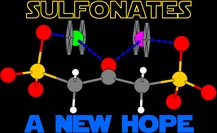

## 1 Introduction

The investigation of organosulfonic acids and their derivatives started already at the end of the 19th century, for example, by *Strecker* [1], *Carius* [2], and *Monari* [3]. Although these acids have been known for over a century, they are still investigated as strong *Brønsted* acids for catalytic applications [4, 5, 6], for functionalization strategies toward pharmaceuticals [7, 8], and have quite recently gained increased interest as materials with nonlinear optical properties (NLO materials) [9, 10, 11]. While a significant number of organosulfonates have been prepared in oleum (SO_3_‐rich H_2_SO_4_) [12], reactions in neat sulfur trioxide SO_3_ [13] and using SO_3_ adducts work as well [14, 15]. Unfortunately, for most of the reported organosulfonic acids and their salts, the respective solid‐state structures are unknown. Therefore, the constitution of many of these species is still unclear [16]. Notably, the structure of the simplest organosulfonic acid, that is, methanesulfonic acid, H_3_CSO_3_H, was unknown until recently, when we were able to resolve the structure using an in situ grown single crystal [17]. The same is true for the solid‐state structures of the higher methanesulfonic acids H_2_C(SO_3_H)_2_ and HC(SO_3_H)_3_. They were known as their hydrates, that is, as their oxonium salts [18, 19], but it was only recently that we reported the structures of the neat acids [20]. The preparation of methanetrisulfonates [HC(SO_3_)_3_]^3–^ is achieved by the reaction of acetone with oleum. Interestingly, the sulfonation of acetone has also been reported to produce acetonesulfonic and acteonedisulfonic acid, respectively [21]. Especially acetonedisulfonic acid, strictly speaking propanone‐1,3‐disulfonic acid, and some of its reactions have been reported by *Grot* in 1965 [22]. However, neither the acid nor examples of its salts have been structurally characterized so far. We believe that knowledge of the structural data of such fundamental compounds is mandatory to understand their properties. In particular, the lack of structural characterization not only inhibits a better understanding of the underlying constitution but also bears the risk of misinterpretation. As we recently could show for adducts between pyridine and SO_3_, the lack of thorough structural elucidation has led to the overlooking of highly interesting structural motifs, even if these had been proposed earlier [23]. In the course of our detailed investigations of the reactions of organic molecules with SO_3,_ we also started to scrutinize the system SO_3_/acetone in more detail. Here, we present for the first time the structural characterization of acetonedisulfonic acid (ADSA, **1**) and some of its salts, accompanied by further analytics and quantum chemical calculations.

## 2 Results and Discussion

### 2.1 Syntheses

**SCHEME 1:**
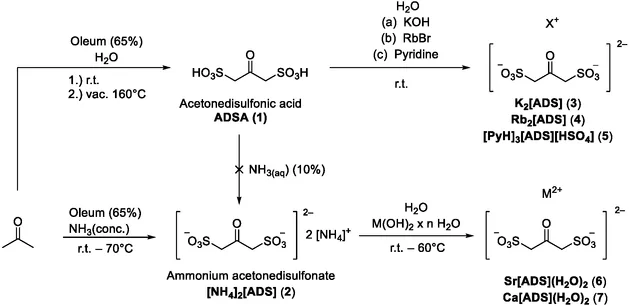
Synthesis of acetonedisulfonic acid (ADSA, **1**), its ammonium salt (**2**), and subsequent neutralization, as well as metathesis reactions thereof.

We found that the preparation of neat acetonedisulfonic acid (**ADSA**, **1**) is possible by slow addition of acetone to oleum (65%), followed by subsequent removal of water from the system (vacuum ~10^−3^ mbar, 160 °C), leading to the crystallization of **1** from the mother liquor (detailed experimental procedures can be found in the Experimental Section). Through this process, a dark‐brown coloration occurs and indicates the formation of pyrolysis products due to unwanted side reactions. The mechanism toward the formation of **1** is most likely an acid‐catalyzed enolization with subsequent sulfonation of the resulting C═C double bond [21, 24, 25]. Nonetheless, we were able to use the prepared ADSA for subsequent neutralizations toward the potassium acetonedisulfonate salt K_2_[ADS] (**3**), according to the procedure by Grot, and advance it toward the novel rubidium salt Rb_2_[ADS] (**4**), as well as the mixed anionic pyridinium (PyH) salt [PyH]_3_[ADS][HSO_4_] (**5**). We did not achieve the ammonolysis reported by *Grot* but observed the formation of [NH_4_]_3_H[SO_4_]_2_ [26]. Alternatively, we present a direct, one‐pot synthesis for the ammonium salt [NH_4_]_2_[ADS] (**2**) from oleum, acetone, and concentrated ammonia, yielding **2** as a phase‐pure crystalline solid (for PXRD with *Rietveld* refinement see Figure S3). All reactions and products are summarized in Scheme 1. Compound **2** can be readily used for salt metathesis reactions, as we show by the exchange of the cation toward the novel strontium (**6**) and calcium compounds (**7**). Neutralization approaches using CsBr and LiBr led to the formation of the hydrogen sulfate [HSO_4_]^‒^ salts, and reactions with Ca(OH)_2_ and MgCO_3_/Mg(OH)_2_ yielded the starting materials. The salt metathesis of **2** does not work with Ba(OH)_2_·8H_2_O, CsNO_3,_ and AgNO_3_. A summary of the unsuccessful reactions is provided in the Supporting Information (Table S51).

### 2.2 Crystal Structure of Acetonedisulfonic Acid, ADSA

**FIGURE 1:**
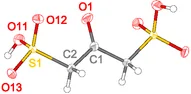
Structure of the acetonedisulfonic acid molecule in the solid state. Selected distances (in pm) and angles (in °) compared to theoretical values (in italics): S1–O11 153.3(2)/*158.7*, S1–O12 143.5(2)/*142.3*, S1–O13 144.7(2)/*142.5*, S1–C2 176.4(2)/*178.0*, C2–C1 152.3(3)/*151.5*, C1–O1 120.1(5)/*119.9*, O11–H1 83.9(5)/*96.7*; S1–C2–C1 112.5(2)/*112.6*, C2–C1–C2´ 115.2(3)/*117.6*. The ellipsoids are drawn at a 50% probability level.

ADSA (**1**) crystallizes with monoclinic symmetry (space group *C*2/*c*) and four formula units in the unit cell. The sp^2^ carbon atom of the carbonyl group is surrounded in an almost perfect trigonal planar manner by a double‐bonded oxygen atom (120.1(5) pm) and two crystallographically identical sp^3^ carbon atoms (152.3(3) pm). The carbon and the oxygen atom of the carbonyl group are located on the special *Wyckoff* position 4*e*, bearing site symmetry 2. Accordingly, the ADSA molecule has *C*_2_ symmetry in the crystal structure with two crystallographically equivalent [HSO_3_] moieties, which show the typical distances of sulfonate groups (cf. Figure 1). The observed distances are well reproduced by density functional theory (DFT) calculations. The orientation of the [HSO_3_] groups with respect to each other occurs in a way that the backbone atoms C1, C2, S1, and O13, as well as the carbonyl oxygen atom O1, are nearly lying on a mirror plane. It is only the difference between the atoms O12 and O11, the OH group of the acid, that impedes a higher *C*_2v_ symmetry for the molecule.

**FIGURE 2:**
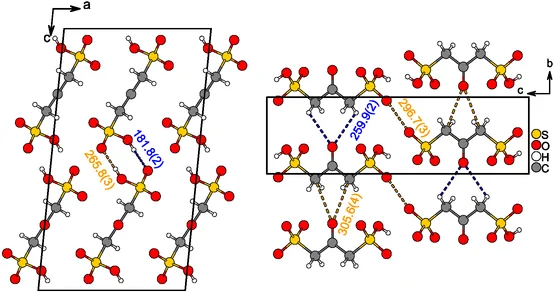
Packing of molecules in the crystal structure of **1**, viewed along [010] (left) and [100] (right). Interatomic distances are given in [pm]. Blue and orange dotted lines indicate the hydrogen bonds (H···X) and donor–acceptor (O···O/C···O) distances, respectively.

The crystal structure of **1** is strongly influenced by the formation of intermolecular hydrogen bonds, as depicted in Figure 2.

Although the donor–acceptor distance in **1** is slightly elongated compared to H_3_CSO_3_H (261.45(9) pm) [17], the O─H─O angle of 178(1)° shows a remarkable directionality, which is why the hydrogen bond can be considered strong [27, 28]. Since every sulfonic acid group provides the donor and acceptor functionality in close proximity to each other, a strand‐like, 1‐D twisting chain is formed along the crystallographic *c*‐axis. For complex hydrogen bonding patterns like this, the “Graph‐Set Analysis” by *Etter* and *Bernstein* can be used for further characterization [29, 30]. The herein shown strand formation belongs to the graph set *R*_2_^2^(8), which is regularly encountered in nature [31]. An additional coordination motif is found if the packing of **1** is viewed along the [100] direction, whereby the [(HOSO_2_CH_2_)_2_CO] units are stacked on top of each other along the *b*‐direction. Although the shown O···O and C(O)···H(CH) contacts either do not share a hydrogen atom or are not classical hydrogen bonds, their close proximity is still within the region of attractive interactions [32].

When moving from the solid‐state structures of **1** toward its salts, the large structural variety of organosulfonates, already known from the salts of H_3_CSO_3_H [33, 34] or HC(SO_3_H)_3_ [19], becomes apparent.

### 2.3 Crystal Structures of Acetonedisulfonates

**FIGURE 3:**
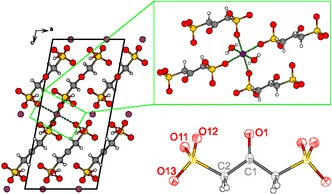
The crystal structure of Ca[ADS](H_2_O)_2_ (**7**, left hand) is very similar to that of the neat acid (cf. Figure 2). Compared to the latter, the anions are connected by octahedral‐coordinated Ca^2+^ ions (upper part, right hand). The [ADS]^2−^ ions display the same conformation as the acid molecules (cf. Figure 1) with almost *C*_2v_ symmetry (lower part, right hand). Also, the distances are very similar to those of the ADSA molecule. Selected distances (in pm) and angles (in °) compared to theoretical values (in italics): S1–O11 144.8(2)/*145.6*, S1–O12 146.4(2)/*145.8*, S1–O13 146.1(2)/*146.2*, S1–C2 176.7(3)/*182.4*, C2–C1 151.7(3)/*150.3*, C1–O1 120.5(5)/*121.3*, S1–C2–C1 116.7(2)/*114.6*, C2–C1–C2´ 110.2(3)/*116.5*. The ellipsoids are drawn at a 70% probability level. Color code: Ca—magenta, S—yellow, O—red, C—gray, H‍—‍white.

As mentioned above, the ADSA molecule exhibits almost *C*_2v_ symmetry, which is only suppressed by the OH groups of the acid. Thus, the respective undisturbed anion should adopt this symmetry. Interestingly, the crystal structure of the calcium salt Ca[ADS](H_2_O)_2_ (**7**) shows the anion in almost the same conformation as that displayed by the acid ADSA (Figure 3, left). The symmetry of the compound (monoclinic, *C*2/*c*) also allows only *C*_2_ symmetry for the anion. The unit cell of **7** is similar to that of the neat acid if a doubling of the *b*‐axis is encountered (Table 1). Furthermore, the *a*‐axis is remarkably shorter due to the strong ionic interactions of the anions and Ca^2+^ ions. For the [ADS]^2–^ anion, the S─O bonds are found quite uniformly between 144.9(2) and 146.4(2) pm, and the remaining distances are almost the same as found for the acid molecule (cf. Figure 1 and Supporting Information). The orientation of the [SO_3_] groups with respect to each other can be judged by the torsion angle S1–C2–C2´–S1´. It is found at 14.2° for the neat acid and at 24.2° for Ca[ADS](H_2_O)_2_, corroborating the strong structural similarity of the anion in the calcium compound and the findings for the acid molecule.

**TABLE 1:** Selected crystallographic data of ADSA and Ca[ADS](H_2_O)_2_.

Compound	[(HO_3_SCH_2_)_2_CO]	Ca[(O_3_SCH_2_)_2_CO](H_2_O)_2_
*T,* K	100	100
Crystal system	*Monoclinic*	*Monoclinic*
Space group	*C*2*/c*	*C*2*/c*
*a*, pm	920.11(5)	762.02(8)
*b*, pm	479.03(2)	804.48(9)
*c*, pm	1645.96(9)	1613.1(2)
*β*, °	95.651(2)	100.540(4)
*V*, Å^3^	721.95(6)	972.2(2)
*Z*	4	4
Final *R* indexes [all data]	*R*_1_ = 0.0484, *wR*_2_ = 0.0936	*R*_1_ = 0.0564, *wR*_2_ = 0.0918
*R*_int_	0.0172	0.0242
*Goof*	1.138	1.152

**FIGURE 4:**
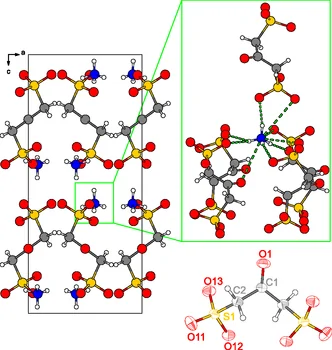
The crystal structure of the salts M_2_[ADS] (M = K, Rb, NH_4_) on the example of the ammonium compound (**2**, left hand). Note, that the Rb salt displays a slight monoclinic distortion even if the structure is almost identical (cf. Table 2). The [ADS]^2–^ ions have a significantly different conformation compared to the structure of **7** and show *C*_2_ symmetry (lower part, right hand). The ninefold coordination of the M^+^ cations (here NH_4_^+^) is shown in the upper right part. The ellipsoids are drawn at a 50% probability level. Color code: N—blue, S—yellow, O—red, C—gray, H—white.

The orientation of the [SO_3_] groups with respect to each other can be different in the [ADS]^2–^ anion due to the possible free rotation around the C─C single bonds. Indeed, the conformation of the anion in the salts M_2_[ADS] (M = K, Rb, and NH_4_) is very different from the findings for the calcium compound. Here, the C═O moiety is also located on a twofold axis, but the torsion angle is found around 130° for all of the three compounds (Figure 4). The crystal structures of the potassium and ammonium salts are orthorhombic, and the rubidium compound is monoclinic. However, the unit cells are very similar, as can be seen from Table 2 (for a direct comparison, the unconventional *Pnca* setting of space group no. 60 should be used). It is worthwhile to mention that the monoclinic [NH_4_]_2_[ADS] undergoes a phase transition between 200 and 160 K, causing twinning of the crystal. Therefore, this compound has been measured at 260 K. With respect to the ionic radii, NH_4_^+^ and Rb^+^ are often isotypic, but there are numerous compounds with (slightly) different crystal structures known, also for oxoanionic compounds, with the [SO_4_]^2–^ and [FSO_3_]^–^ salts being prominent examples [35, 36, 37, 38]. The M^+^ cations (M = K, Rb, NH_4_) show a ninefold coordination of oxygen atoms, which belong to two monodentate and three chelating [ADS]^2–^ anions. Interestingly, the oxygen atom of the C═O groups also takes part in the coordination.

**TABLE 2:** Selected crystallographic data of M[ADS] (M = K, Rb, NH_4_).

Compound	K_2_[(O_3_SCH_2_)_2_CO]	[NH_4_]_2_[(O_3_SCH_2_)_2_CO]	Rb_2_[(O_3_SCH_2_)_2_CO]
*T,* K	100	260	100
Crystal system	*Orthorhombic*	*Orthorhombic*	*Monoclinic*
Space group	*Pbcn*	*Pbcn*	*P*2_1_/*c*
*a*, pm	730.44(4)	747.38(3)	1701.40(9)
*b*, pm	739.99(4)	763.44(4)	752.06(3)
*c*, pm	1637.8(1)	1713.59(9)	757.14(4)
*β*, °	90	90	94.562(2)
*V*, Å^3^	885.26(9)	977.74(8)	965.73(8)
*Z*	4	4	4
Final *R* indexes [all data]	*R*_1_ = 0.0319, *wR*_2_ = 0.0640	*R*_1_ = 0.0316, *wR*_2_ = 0.0825	*R*_1_ = 0.0290, *wR*_2_ = 0.0756
*R*_int_	0.0319	0.0502	0.0450
*Goof*	1.118	1.105	1.091

**FIGURE 5:**
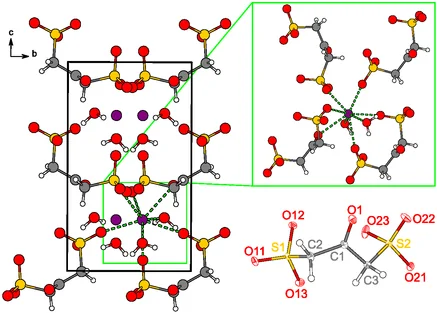
The crystal structure of the salts Sr[ADS](H_2_O)_2_ (**5**, left‐hand). The [ADS]^2−^ ions have a significantly different conformation compared to the structure of **7** and show *C*_1_ symmetry (lower part, right hand). The eightfold coordination of the Sr^2+^ cation is shown in the upper right part. The ellipsoids are drawn at a 50% probability level. Color code: Sr—magenta, S—yellow, O—red, C—gray, H—white.

Another possible conformer of the [ADS]^2–^ anion is found in the strontium salt, Sr[ADS](H_2_O)_2_. Here, the anion has only *C*_1_ symmetry, and the torsion angle S1–C2–C3–S3 shows a value of 54.7° (Figure 5). The non‐centrosymmetric anions are oriented in the same way within the orthorhombic crystal structure: one of the [SO_3_] groups lies in the same plane as the carbon backbone of the anion, and the second sulfonate group points in the [001] direction. Thus, the resulting structure also does not contain a center of symmetry, as reflected by the non‐centrosymmetric space group *Pca*2_1_. The Sr^2+^ ions are in a typical eightfold coordination of oxygen atoms, with the crystal water molecules acting as additional ligands. Crystallographic details are given in Table 3.

**TABLE 3:** Selected crystallographic data of Sr[ADS](H_2_O)_2_ and [PyH]_3_[ADS][HSO_4_].

Compound	Sr[(O_3_SCH_2_)_2_CO](H_2_O)_2_	[H_6_C_5_N]_3_[(O_3_SCH_2_)_2_CO][HSO]_4_
*T,* K	100	100
Crystal system	*Orthorhombic*	*Orthorhombic*
Space group	*Pca*2_1_	*Pca*2_1_
*a*, pm	1087.7(2)	1909.4(3)
*b* pm	725.3(1)	1017.7(2)
*c*, pm	1222.6(2)	1167.9(2)
*V*, Å^3^	964.5(3)	2269.4(7)
*Z*	4	4
Final *R* indexes [all data]	*R*_1_ = 0.0211, *wR*_2_ = 0.0449	*R*_1_ = 0.0892, *wR*_2_ = 0.1653
*R*_int_	0.0540	0.01128
*Goof*	1.068	1.093

**FIGURE 6:**
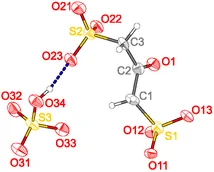
Structure and labeling of the acetonedisulfonate and the hydrogensulfate anion in [PyH]_3_[ADS][HSO_4_]. Selected distances (in pm) and angles (in °): S1–O11 144.4(7), S1–O12 145.2(7), S1–O13 146.3(7), S1–C1 178.3(1), C2–C1 151.5(1), C2–C3 150.7(1), C2–O1 120.3(1), S2–O21 144.5(7), S2–O22 145.7(7), S2–O23 145.7(7), S2–C3 178.8(1), S3–O31 144.0(8), S3–O32 144.2(8), S3–O33 144.8(7), S3–O34 159.8(7), O34–H1 83.9(5), O23–O34 264.5(7); S1–C2–C1 115.3(7), O34–H1–O23 160.5(2). The ellipsoids are drawn at a 50% probability level.

In the pyridinium salt [PyH]_3_[ADS][HSO_4_] (crystallographic details are given in Table 3), the conformation of the [ADS]^2−^ anion is similar to the findings for the M^+^ salts (M = K, Rb, NH_4_) discussed above, with a torsion angle S1–C1–C3–S2 of 122.3°. A specialty found in this compound is the presence of a second anion, namely, [HSO_4_]^‒^. Both ions are connected by a short hydrogen bond with a donor–acceptor distance between the atoms O34 and O23 of 264.7(3) pm and a bond angle O34‐H1····O23 of 160.5(2)° (see Figure 6).

### 2.4 Comparison of ADSA and Acetonedisulfonate Anions

**FIGURE 7:**
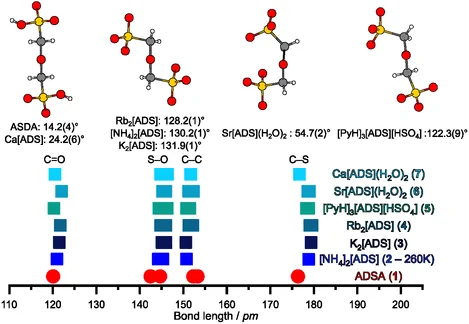
The upper part of the figure shows the different conformations of the acetonedisulfonate ions (and the neat acid) viewed along the O═C bond of the keto group. The torsion angles S─C─C─S include the [CH_2_] groups of the carbon backbone of the anion and can be seen as a measure of the orientation of the [SO_3_] groups of the anion. In the lower part of the figure, the observed distances of characteristic bonds are summarized. The free acid (**1**) is shown as circles, and the derived anions are shown as squares, respectively.

Although the conformation of the [ADS]^2–^ anions might be different, the essential bonding parameters within the anions are quite similar (Figure 7). In the neat acid, [(HO_3_SCH_2_)_2_CO] (ADSA, **1**), the C═O, S─O and C─S bonds are shortened compared to the anionic species. Upon deprotonation, the negative charge delocalizes over the SO_3_ unit, leading to an elongation of the S─O bonds and, due to the removal of electron density from the central sulfur atom, increasing C─S bonds.

The C═O bond within **1** (120.1(5) pm) is slightly shortened compared to unsubstituted acetone (121.0(3) pm), whereas the C─C bond length (152.3(3) pm) is significantly elongated (148.6(3) pm) [39]. This is expected to be due to the strong electron withdrawal introduced by the sulfonic acid groups. The C─S bond lengths in **1** (176.4(2) pm) are moderately elongated compared to H_3_CSO_3_H (173.8(1) pm), while the respective S─O distances are in perfect agreement due to the fact that both acids form strong hydrogen bonds [17]. This is also found within the oxonium salts of strong organosulfonic acids, e.g., [H_3_O]_2_[H_2_C(SO_3_)_2_] or [H_3_O]_3_[HC(SO_3_)_3_] [18, 19]. However, the C─S bond elongation is less pronounced than for the methanedisulfonic acid (178.14(4) pm) since the carbonyl group is less electron‐withdrawing compared to another sulfonic acid moiety [20]. The bond lengths within compounds **2**–**7** show the general trends known from polysulfate chemistry, say, an increasing S─O bond length with increasing “hardness” of the cation (NH_4_/K < Rb, Sr < Ca) [40, 41, 42, 43]. Additionally, different coordination motifs around the cations and different denticities of the [ADS]^2–^ anions can be found, as shown in Figures 3–5. For the geometrical analysis of the coordination polyhedra and their deviation from the ideal geometry, the software *Polynator* was used [44]. The resulting *δ* values provide insight into the coordination geometry around the central cation, where larger values indicate a greater deviation from the ideal geometry. We consider *δ* values up to 20 as still suitable geometric descriptions. While the potassium compound **3** (coordination number (CN) = 9, no regular polyhedron) shows a coordination number regularly encountered for oxoanionic potassium compounds (CN = 6–10) [19, 45, 46, 47, 48], compounds **4**, **6,** and **7** differ. Although only a few (metal) organosulfonate structures are available for comparison, the coordination number for the rubidium compound **4** (CN = 9, no regular polyhedron) differs compared to the simple sulfate (CN = 10) [49] and the methanesulfonate solvate (CN = 8) [50] but is in good agreement with the methane(poly)sulfonate salts [20]. The coordination for strontium in compound **6** (CN = 8, tetragonal antiprism, *δ*_TA_ = 7.457) is increased compared to the known methanesulfonate and trisulfate [40, 51]. The most surprising coordination is the distorted octahedron of calcium compound **7** (*δ*_O_ = 4.720), something rather rarely encountered despite the mass of inorganic and organic Ca^2+^ salts [52, 53]. For the methanolic solvation of the Ca^2+^ cation, a sixfold coordination was calculated to be energetically most favorable [54]. Besides the unusual metal‍–‍anion coordination, compounds **2** and **5**, having accessible hydrogen‐bond donors as their charge‐stabilizing cations, show remarkable coordination motifs. To better understand these systems, we therefore additionally applied quantum chemical calculations to quantify the strength of the present noncovalent interactions.

### 2.5 Quantum Chemical Calculations

**FIGURE 8:**
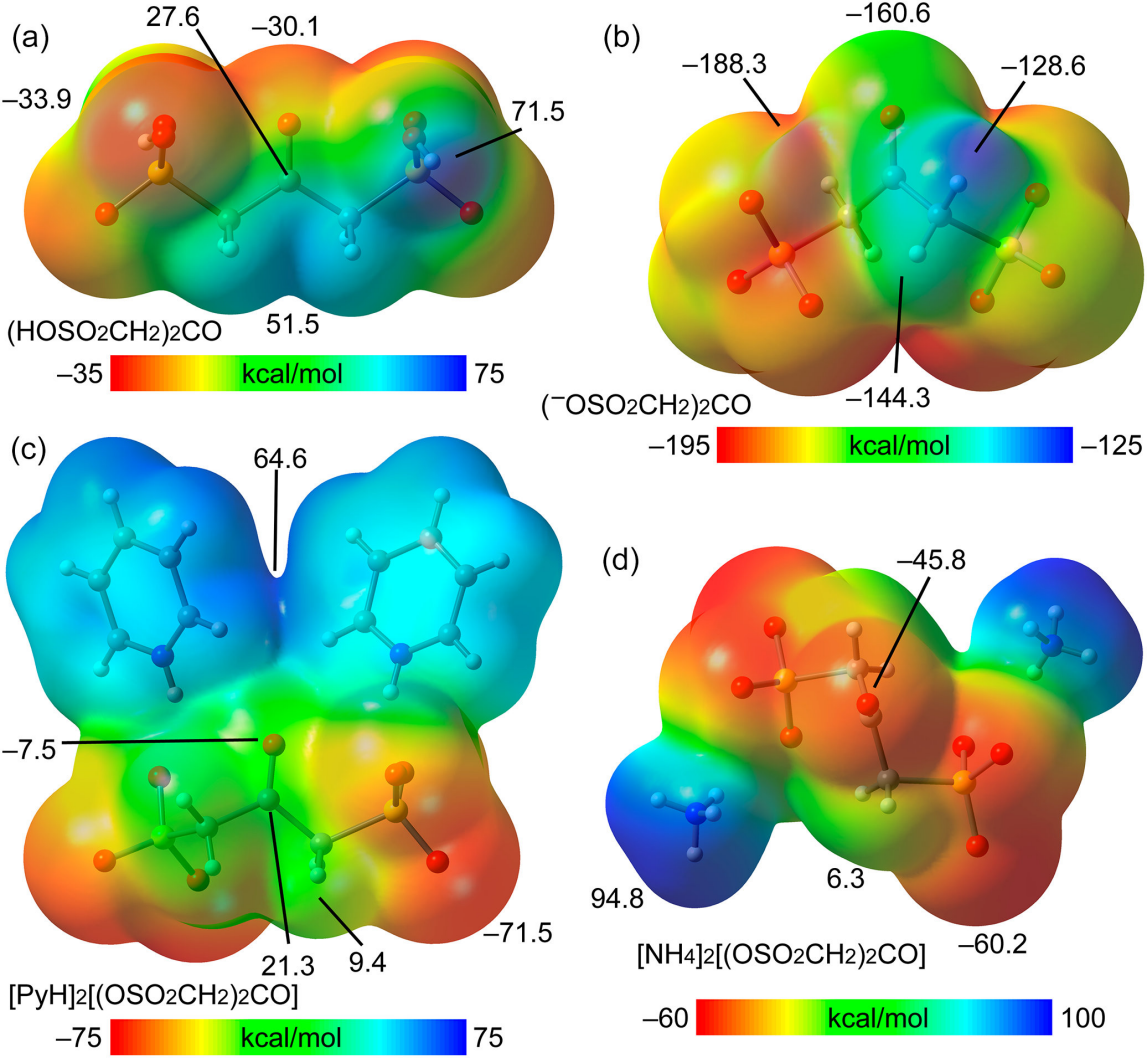
MEP surfaces of (a) **1**, (b) acetonedisulfonate dianion, (c) **5**, and (d) **2** (isosurface 0.001 a.u.). The electrostatic potential values at specific points are provided in kcal/mol. The color scale for each plot ranges from red negative to blue positive according to the bars shown for each individual system.

To investigate the electronic properties and the nature of the noncovalent interactions within the crystal structures, DFT calculations were conducted. The structures of the salts **2**, **3**, **4**, **6,** and **7** are certainly dominated by Coulombic interactions. However, ammonium salt **2** and pyridinium compound **5**, as well as neutral acid **1,** are characterized by hydrogen–bonding interactions. Consequently, the theoretical study targets the molecular electrostatic potential (MEP) surfaces and the quantification of hydrogen‐bond strengths in these three specific systems to provide a consistent comparison of their electronic landscapes. The MEP surfaces were computed for the neutral acid, the dianion, and salts **2** and **5** to examine their electronic distribution and identify potential reactive sites (Figure 8). For the neutral acid (Figure 8a), the global maximum is found at the acidic protons of the sulfonic acid groups (71.5 kcal/mol), while the global minimum is located at the sulfonic oxygen atoms (–33.9 kcal/mol). The keto oxygen atom shows a significant negative potential (–30.1 kcal/mol), and the keto carbon atom exhibits a positive potential (27.6 kcal/mol). Notably, the methylene protons display a high positive potential (51.5 kcal/mol), which is consistent with their enhanced acidity due to the alpha‐position relative to both the carbonyl and sulfonic acid groups. This is further corroborated by the spectroscopic data (NMR and IR, see Figures S10–S14 and Table S50). The IR spectrum can be well fitted to the calculated spectrum of the [ADS]^2–^ spectrum when applying a scale factor of 0.956 and shows well‐resolved, characteristic normal modes, extending the literature report [22]. The observed normal modes are in good agreement with the original data by *Grot* *,* and the broad peaks between 3064 and 3215 cm^–1^ already indicate that the ammonium cation engages in strong hydrogen bonding. Both ^1^H and ^13^C‐NMR spectra as well as 2D spectra show the expected sets of singlets and their correlations.

In contrast, for the dianion (Figure 8b), all MEP values are negative, as expected for a doubly charged species. The global minimum is situated at the sulfonate oxygen atoms (–188.3 kcal/mol), followed by keto oxygen (–160.6 kcal/mol). The methylene hydrogen atoms represent the local maxima, with values ranging from –128.6 to –144.3 kcal/mol. This stark contrast highlights the tunable reactivity upon protonation/deprotonation of the sulfonic acid groups. Whereas a nucleophilic attack at the carbonyl carbon seems possible for the free acid (**1**), the deprotonation of the sulfonic acid groups will greatly inhibit this reactive center.

The MEP surfaces for the pyridinium and ammonium surfaces (Figure 8c,d) show minima at the anionic units (–71.5 kcal/mol for the pyridinium salt and –60.2 kcal/mol for the ammonium salt) and maxima at the cations (64.6 kcal/mol for pyridinium and 94.8 kcal/mol for ammonium). The methylene protons in these salts remain positive (9.4 and 6.3 kcal/mol, respectively). Interestingly, the keto carbon atom is positive in the pyridinium salt (21.3 kcal/mol), whereas it is not accessible in the ammonium salt. Furthermore, the keto oxygen atom in the ammonium salt is much more negative (–45.8 kcal/mol) than that in the pyridinium salt (–7.5 kcal/mol), suggesting different degrees of electronic stabilization or steric shielding by the respective cations in these models. This further corroborates the aforementioned possibility of altering the reactivity, not only by protonation/deprotonation but also by changing the respective counter ion.

**FIGURE 9:**
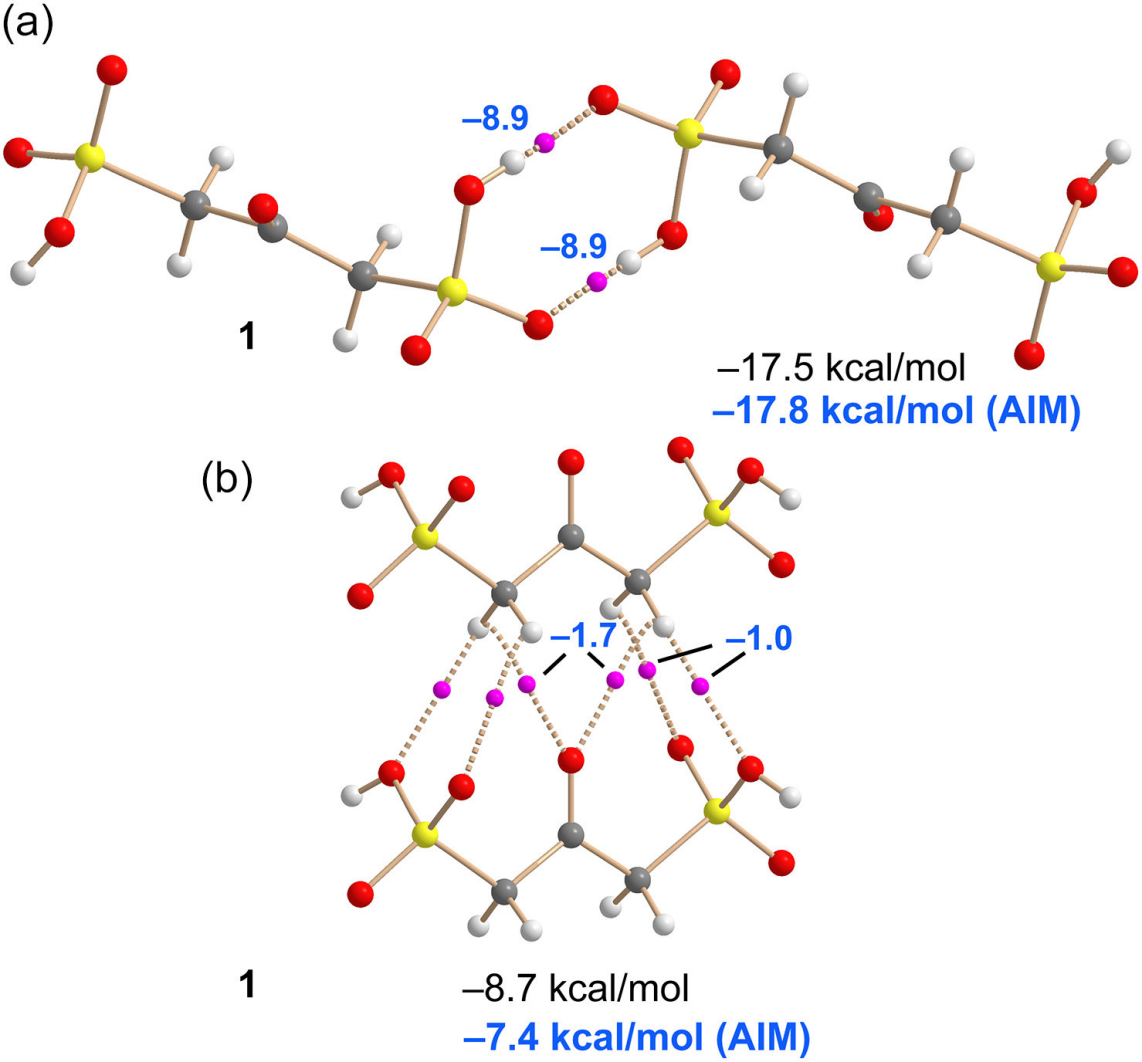
Energetic and QTAIM analysis (BCPs in pink and bond paths as dashed bonds) of two supramolecular dimers extracted from the X‐ray crystal structure of **1**. (a) Self‐assembled dimer featuring a central *R*_2_^2^(8) hydrogen‐bonded synthon which propagates into 1D infinite chains. (b) Alternative supramolecular dimer stabilized by cooperative CH···O contacts involving the keto group and methylene protons. Energies are provided in kcal/mol. Black text indicates computed dimerization energy; blue text indicates QTAIM‐predicted energies (AIM) for the dimer total and individual bonds.

To elucidate the supramolecular architecture of **1**, both energetic and *Bader’s* quantum theory of atoms in molecules (QTAIM) analyses were performed on two different dimeric motifs extracted directly from its experimental X‐ray structure (Figure 9).

Figure 9a depicts the assembly along the crystallographic *a*/*c*‐axes: a self‐assembled dimer that forms strong *R*_2_^2^(8) hydrogen‐bonded synthons between adjacent molecules [29, 30]. These motifs serve as the foundational units that further propagate to form infinite 1D chains in the solid state. The computed dimerization energy for this assembly is remarkably large (–17.8 kcal/mol), underscoring its pivotal importance in the structure of **1**. Furthermore, QTAIM analysis reveals bond critical points (BCPs, represented as pink spheres) corresponding precisely to these hydrogen–bond interactions. The hydrogen bond strength was calculated from the potential energy density at these points using the equation proposed by Espinosa et al. [55], (*E* = 0.5 × *V*(*r*)), yielding values of –8.9 kcal/mol for each individual bond (indicated in blue). This sums up to –17.8 kcal/mol, showing that the values are virtually identical to the purely energetic calculation and confirming QTAIM as a highly reliable tool for predicting the energy of these specific interactions.

In Figure 9b, the stacking motif of **1** along the crystallographic *b*‐axis is analyzed. In this configuration, the keto group, working cooperatively with the sulfonic oxygen atoms, establishes a network of C─H···O contacts involving the acidic methylene protons, in line with the MEP analysis. This dimeric unit propagates in a head‐to‐tail fashion, resulting in the formation of an infinite 1D assembly throughout the crystal structure. The computed dimerization energy for this interaction is –8.7 kcal/mol, thus considerably less modest than the energy of the *R*_2_^2^(8) dimer. The energy predicted via QTAIM (–7.4 kcal/mol) is slightly smaller, yet in reasonable agreement with the standard energy calculation, confirming that the weaker interaction still contributes to the overall cohesion of the crystal.

**FIGURE 10:**
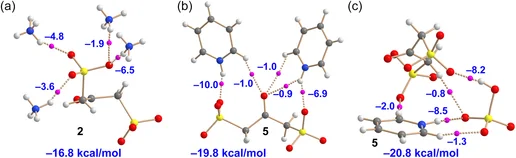
QTAIM analysis of the hydrogen‐bond assemblies in salts **2** and **5**. (a) Interaction between a sulfonate group and [NH_4_]^+^ cations in salt **2**. (b) Interaction of keto and sulfonic groups with pyridinium cations in salt **5**. (c) Trimeric assembly in salt **5** involving a pyridinium cation, an acetonedisulfonate dianion, and an [HSO_4_]^‒^ unit. All energies are provided in kcal/mol and correspond to the potential energy density at the bond critical points. Small pink spheres indicate bond critical points BCPs.

To evaluate the individual hydrogen bond strengths independently of the dominant ion‐pair coulombic effects, the QTAIM analysis was extended to the organic salts (Figure 10).

In ammonium salt **2**, the interaction of a sulfonic acid group with four surrounding [NH_4_]^+^ cations was examined (Figure 7a). The directional hydrogen bonds exhibit energies ranging from –1.9 to –6.5 kcal/mol, with a total assembly energy of –16.8 kcal/mol. These individual interactions are weaker than the hydrogen bonds characterized in the *R*_2_^2^(8) homodimer of the neutral acid, indicating that the neutral synthon is inherently more robust once purely coulombic attractions are isolated.

For the pyridinium salt **5**, two distinct supramolecular assemblies were analyzed. The first (Figure 7b) involves the interaction of the keto and sulfonic acid groups with two pyridinium cations via two NH···O(sulfonate) and three CH···O(keto) hydrogen bonds. In agreement with the MEP analysis, the NH···O bonds are significantly stronger (–6.9 and –10.0 kcal/mol) than the CH···O contacts (approximately –1.0 kcal/mol each), resulting in a total assembly energy of –19.8 kcal/mol. The second assembly is a trimer (Figure 10c) composed of one pyridinium cation, one acetonedisulfonate anion, and one [HSO_4_]^‒^ unit. Within this cluster, the two anions are interconnected by a weak CH···O contact (–0.8 kcal/mol) and a strong OH···O H‐bond (–8.2 kcal/mol). The pyridinium cation serves as a bridge, establishing a CH···O contact (–2.0 kcal/mol) with the acetonedisulfonate and two H‐bonds with the [HSO_4_]^‒^ unit (one strong NH···O with –8.5 kcal/mol and a weaker contact with –1.3 kcal/mol). The total interaction energy for this trimeric assembly is –20.8 kcal/mol. This QTAIM‐based partitioning is essential for identifying the specific contacts, including anion–anion interactions, that are most relevant in dictating the solid‐state architecture of these organic salts.

## 3 Conclusion

We showed that acetonedisulfonic acid (**ADSA**, **1**) is directly accessible from the reaction of acetone and oleum. To avoid unwanted side products due to pyrolysis, we showed that the preparation of the ammonium salt [NH_4_]_2_[ADS] (**2**) from a one‐pot reaction is possible and that the salt can be used for subsequent metathesis reactions. We presented an in‐depth structural analysis of the solid‐state structures of seven compounds. The [ADS]^2–^ dianion displays various conformations, proving the flexibility of the anion and its structural versatility. DFT analysis confirmed the electrophilicity of the keto‐carbon atom and the nucleophilic character of the keto‐oxygen atom, alongside the high acidity of the hydrogen atom of the sulfonic acid group and a remarkable acidity of the methylene protons of **1**. The supramolecular study identified a clear hierarchy of interactions, where the neutral acid is primarily stabilized by robust *R*_2_^2^(8) synthons. A QTAIM partitioning revealed that these neutral hydrogen bonds are inherently stronger than the individual N─H···O interactions in **2**, if purely coulombic effects are excluded. The excellent agreement between the total interaction energies and the QTAIM predictions validates this methodology for characterizing hydrogen bonding in sulfonic acid systems. These results not only explain the observed crystal packing but also provide a quantitative framework for assessing the stability of future organic sulfonic acid derivatives.

## 4 Experimental Section

If not stated otherwise within the respective procedure, all chemicals were used as received without further purification. Deionized water was used if not stated otherwise.

### 4.1 Synthesis of [(HO_3_SCH_2_)_2_CO] (1)

In an oven‐dried 50 mL *Schlenk* tube, 12.5 mL (202.0 mmol SO_3_, 3.8 eq.) fuming sulfuric acid (65% SO_3_, Merck, Darmstadt, Germany) was cooled to –10 °C (ice/EtOH). Through a septum, 3.9 mL (53.1 mmol, 1.00 eq.) of acetone (technical grade) was added in small portions (~2 mL/h) to avoid overoxidation (the mixture should remain colorless). After the addition was completed, the mixture was stirred at room temperature overnight, whereby solidification occurred. Water was added until the mixture dissolved completely, whereby a color change to light brown occurred. The solution was dried in vacuo, leading to the formation of a brown crystalline slurry, from which single crystals suitable for single‐crystal X‐ray diffraction (SCXRD) could be isolated. The product was used for the following reactions without further purification. The crystals are hygroscopic and dissolve upon storage in a moist atmosphere.

### 4.2 Synthesis of [NH_4_]_2_[(O_3_SCH_2_)_2_CO] (2)

In an oven‐dried 50 mL *Schlenk* tube, 25 mL (403.9 mmol SO_3_, 3.8 eq.) fuming sulfuric acid (65% SO_3_, Merck, Darmstadt, Germany) was cooled to –10 °C (ice/EtOH). Through a septum, 7.8 mL (106.1 mmol, 1.0 eq.) of acetone (technical grade) was added in small portions (~2 mL/h) to avoid overoxidation. The mixture solidified after ~1.5 mL of acetone was added and redissolved upon further addition. After the complete addition, the mixture was stirred for 12 h, heated to 70 °C, and stirred for another 4 h. Subsequently, the reaction was quenched with 20 mL of H_2_O and neutralized with conc. NH_3_ until no further gas evolution was observed. Activated charcoal was added, and the mixture was filtered over a paper filter and dried in vacuo, yielding 7.67 g of [NH_4_]_2_[(O_3_SCH_2_)_2_CO]. Upon storage of the mother liquor at 7 °C, additional large single crystals were grown within 1 week, yielding an additional 4.29 g of **2** (total yield 44.76%).

^**1**^ **H‐NMR** (DMSO, 300 MHz) *δ* [ppm] = 7.09 (8H, s, NH_4_), 3.85 (4H, s, CH_2_).

^**13**^ **C‐NMR** (DMSO, 75 MHz) *δ* [ppm] = 195.46 (C═O), 63.83 (CH_2_).

**FT‐IR** *ν* [cm^–1^] = 3219 + 3057 (m, N─H), 1697 (m, C═O), 1412 (s, *ν*_as_ S─O), 1180 (s, *ν*_as_ S–O), 1021(s, *ν*_s_ S─O), 799 (m, *ν* C─S), 676 + 568 + 520 (s, *δ* S─O).

### 4.3 Synthesis of K_2_[(O_3_SCH_2_)_2_CO] (3)

0.2 g (0.9 mmol, 1.0 eq.) **1** was dissolved in 5 mL of water and neutralized with KOH (Fisher Scientific) until a slightly basic pH (~8–9) was reached. After slow evaporation of the water, a small amount of colorless crystals suitable for SCXRD was obtained.

### 4.4 Synthesis of Rb_2_[(O_3_SCH_2_)_2_CO] (4)

0.2 g (0.9 mmol, 1.0 eq.) **1** was dissolved in 5 mL of water, and a stoichiometric amount of RbBr (Alfa Aesar) (0.15 g, 0.9 mmol, 1.0 eq.) was added. was added under stirring. After slow evaporation of the water, a small amount of colorless crystals suitable for SCXRD was obtained.

### 4.5 Synthesis of [H_6_C_5_N]_3_[(O_3_SCH_2_)_2_CO][HSO_4_] (5)

0.2 g (0.9 mmol, 1.0 eq.) **1** was dissolved in 5 mL of water, and 3 mL of pyridine (Fisher Scientific) (2.95 g, 3.7 mmol, 4.0 eq.) was added under stirring. After slow evaporation of the volatiles, a small amount of colorless crystals suitable for SCXRD was obtained.

### 4.6 Synthesis of Sr[(O_3_SCH_2_)_2_CO](H_2_O)_2_ (6)

0.25 g (1.0 mmol, 1.0 eq.) of **2** was suspended in 3 mL of water, and a stoichiometric amount of Sr(OH)_2_·8H_2_O (Sigma–Aldrich) (0.27 g, 1.0 mmol, 1.0 eq.) was added under stirring. The mixture was heated to 60 °C within 15 min and filtered hot over a paper filter. After slow evaporation of the water, a small amount of colorless crystals suitable for SCXRD was obtained.

### 4.7 Synthesis of Ca[(O_3_SCH_2_)_2_CO](H_2_O)_2_ (7)

0.25 g ( 1.0 mmol, 1.0 eq.) of **2** was suspended in 3 mL of water, and a stoichiometric amount of Ca(OH)_2_ (Alfa Aesar) (0.1 g, 1.0 mmol, 1.0 eq.) was added under stirring. The mixture was heated to 60 °C within 15 min and filtered hot over a paper filter. After slow evaporation of the water, a small amount of colorless crystals suitable for SCXRD was obtained.

### 4.8 Structure Determination and Crystallographic Details

The single‐crystal structure determination was performed on a *Bruker D8 VENTURE KAPPA* diffractometer with a microfocus sealed tube using a multilayer mirror as the monochromator and a *Bruker PHOTON III* detector. As characteristic X‐ray radiation, MoK_α_ (*λ* = 71.073 pm) was used for the measurements. Before the measurements, the crystals were prepared in perfluorinated ether oil (Fomblin YR‐180) and selected using a light microscope equipped with an polarization filter. A suitable single crystal was fixed on a MiTeGen micromount (150 µm polymer loop) and adjusted to the X‐ray beam and cooled down to 100 K in a stream of N_2_. The images with the intensity data were processed using software *APEX5* [56]. The frames were integrated with the Bruker *SAINT* software package using a narrow‐frame algorithm. Absorption effects were corrected using SADABS for multi‐scan absorption correction [57, 58]. The structure solution and refinement were done with software *OLEX2* [59]. Dual methods using *SHELXT* were used for structure solution, and full‐matrix least‐squares methods against *F* ^2^ using *SHELXL* were used for refinement [60, 61]. All illustrations of the crystal structures were made with the program *Diamond 4* using the .cif as the input file [62].

### 4.9 Powder X‐Ray Diffraction and Rietveld Refinement

P‐XRD data were collected on a *Stoe* Stadi‐P powder diffractometer with a *Mythen* detector and a Debye−Scherrer geometry using Mo‐K_α1_ radiation of *λ* = 0.70930 Å. The samples were ground and sealed in glass capillaries with a 0.5 mm diameter. The data were recorded in a range of 2*θ* = 0°–60° within 10.5 h. The Rietveld refinement was performed using the software *TOPAS‐Academic* (*64 V6*) [63, 64]. The refinement process included the scale factor and lattice parameters, along with corrections for zero shift, specimen displacement, and background. Peak profiles were described using the pseudo‐Voigt function of Thompson, Cox, and Hastings [65], with the profile shape parameters *u*, *v*, *w*, and *y* refined accordingly.

## 5 Spectroscopic Investigations

### 5.1 Fourier Transform Infrared Spectroscopy (FT‍‐‍IR) Measurements

All measurements were performed at room temperature under an argon atmosphere in a glove box with a *Bruker Alpha II* device using the *OPUS* software [66]. The Absorption bands are given in the corresponding wavenumber $\tilde{\text{ν}}$ (cm^–1^). The data were plotted using the software *OriginPro* [67].

### 5.2 Nuclear Magnetic Resonance (NMR) Measurements

All NMR experiments were performed at the Inorganic Chemistry Institute of the University of Cologne. Spectra for purity control were recorded on a *Bruker Avance I 300* (^1^H: 300 MHz, ^13^C: 75 MHz) device. For the preparation, ~5–20 mg of the sample was dissolved in ~0.6 mL of a suitable, deuterated solvent in a borosilicate NMR tube. The chemical shift (*δ*) was referenced to the solvent signal (DMSO‐d_6_). ^13^C spectra were recorded with ^1^H‐proton decoupling. The spin–spin coupling was abbreviated with *s* (singlet), *d* (doublet), *dd* (doublet of doublets), *t* (triplet), *dt* (doublet of triplets), *q* (quartet), and *m* (multiplet). The raw data were processed using the software *MestReNova* [68].

## 6 Quantum Chemical Computations

The quantum chemical computations were performed using the Gaussian 16 software package (Revision C.01) [69]. To preserve the experimental supramolecular configurations, crystal coordinates from X‐ray diffraction were used without further geometry optimization. The electronic structure was resolved at the PBE0‐D3/def2‐TZVP level of theory, and vibrational frequencies for the [(O_3_SCH_2_)_2_CO]^2–^ anion were calculated accordingly [70, 71, 72]. The calculated bond lengths were based on geometry optimizations of **1**, [(O_3_SCH_2_)_2_CO]^2–,^ and **5**, respectively. The PBE0 functional, combined with D3 dispersion corrections, provides the accuracy required to quantify both strong directional NH···O and OH···O bonds and weaker ancillary interactions. MEP maps were generated at the same level of theory to visualize the electron density distribution. Topological analysis of the electron density was conducted according to Bader’s QTAIM [73]. Wavefunction files from Gaussian were analyzed with AIMAll software (version 19.10.12) [74]. Bond critical points (BCPs), bond paths, and potential energy density (*V*) values at these BCPs were determined for all relevant hydrogen bond contacts. Finally, interaction energies (*E*) for discrete hydrogen bonds were estimated using the empirical correlation by Espinosa et al. [55]. This relationship links the potential energy density at the BCP with the interaction energy through the formula *E* = 0.5 × *V*, providing a reliable measure of hydrogen bond strength based on QTAIM descriptors.

## Author Contributions

J.L. and M.S.W. designed the project. J.L. and J.C. synthesized and characterized the compounds. A.F. and J.L. planned and conducted the theoretical studies. J.L. wrote the draft of the manuscript. All authors participated in preparing the final manuscript.

## Funding

This work was supported by the Ministerio de Ciencia, Innovación y Universidades (PID2023‐148453NB‐I00).

## Conflicts of Interest

The authors declare no conflicts of interest.

## Supporting information

Supplementary Material

## Data Availability

All data used in this article are available from the Supporting Information file or the Cambridge Structural Database.
