# Supplementary material for: The Sulfonation of Acetone: Characterization of Acetonedisulfonic Acid and Its Salts
Source: Chempluschem. 2026 Sep 7;91(9):e70237. doi: 10.1002/cplu.70237 (PMC13549473; doi:10.1002/cplu.70237)
Supplement: Supplementary file 1 [file CPLU-91-e70237-s001.pdf]

The Sulfonation of Acetone: Characterization of Acetonedisulfonic Acid and its Salts

Jan Langwald,[a] Jan Chrubasik,[a] Antonio Frontera*[b] and Mathias S. Wickleder*[a]

[a] *Institute of Inorganic and Materials Chemistry, University of Cologne, Greinstr. 6, 50939 Cologne, Germany. E-mail: mathias.wickleder@uni-koeln.de*

[b] *University of the Balearic Islands, Department of Chemistry, Institute of Organic Chemistry Carretera de Valldemossa km 7.5, Palma de Mallorca, Spain.*
E-mail: toni.frontera@uib.es

Table of Contents

$[(HO_3SCH_2)_2CO]$ (1) 2

$[NH_4]_2[(O_3SCH_2)_2CO]$ (2) 5

$K_2[(O_3SCH_2)_2CO]$ (3) 8

$Rb_2[(O_3SCH_2)_2CO]$ (4) 11

$[H_6C_5N]_3[(O_3SCH_2)_2CO][HSO]_4$ (5) 15

$Sr[(O_3SCH_2)_2CO](H_2O)_2$ (6) 20

$Ca[(O_3SCH_2)_2CO](H_2O)_2$ (7) 24

Spectroscopic Data 27

Unsucessful Reactions 33

[$(HO_3SCH_2)_2CO$] (1)

Table S1: Crystallographic data of [$(HO_3SCH_2)_2CO$].

Empirical formula	$C_3H_6O_7S_2$
Formula weight	218.20 g/mol
Temperature	100.00 K
Crystal system	Monoclinic
Space group	*C*2/*c* (No. 15)
Unit cell dimensions	a = 920.11(5) pm<br>b = 479.03(2) pm<br>c = 1645.96(9) pm<br>α = 90°<br>β = 95.651(2)°<br>γ = 90°
Volume	721.95(6) $Å^3$
Z	4
ρ_{calc}	2.007 g/cm^3
μ	0.737 mm^{-1}
F(000)	448
Radiation	MoK_α (λ = 0.71073 nm)
Crystal size	0.16 x 0.11 x 0.05 mm^3
2Θ range for data collection	4.974 to 57.398
Index ranges	$-12 \leq h \leq 12$, $-6 \leq k \leq 6$, $-22 \leq l \leq 22$
Reflections collected	6268
Independent reflections	913 [R_{int} = 0.0172, R_σ = 0.0111]
Completeness	98.2%
Absorption correction	multiscan
Min. and max. transmission	0.660 / 0.747
Data/restraints/parameters	913/0/57
Goodness-of-fit on F^2	1.138
Final R indexes [$I \geq 2\sigma$ (I)]	R_1 = 0.0481, wR_2 = 0.0909
Final R indexes [all data]	R_1 = 0.0484, wR_2 = 0.0936
Largest diff. peak/hole	0.53/–0.41 e · $Å^{-3}$
CCDC-No.	2517625

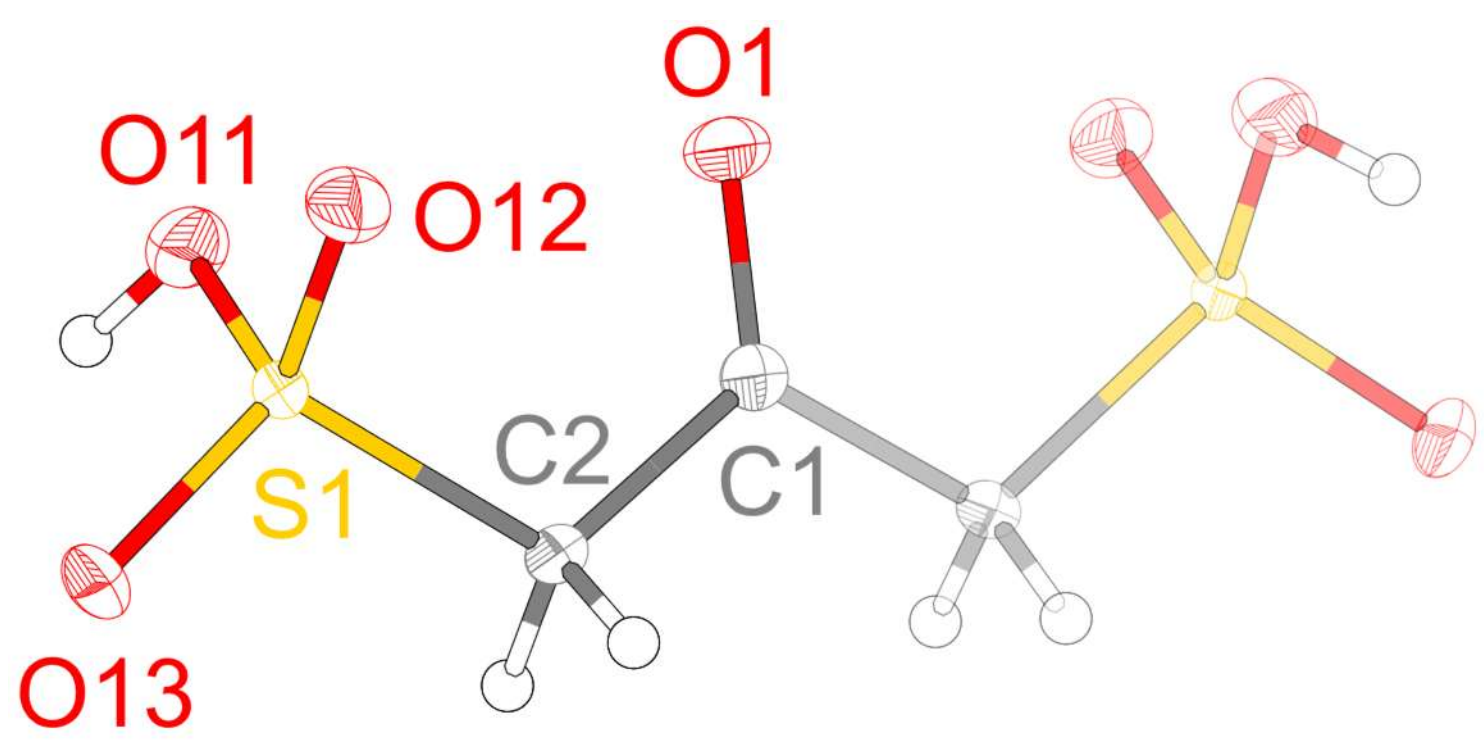

Figure S1: Thermal ellipsoid plot of the asymmetric unit of [$(HO_3SCH_2)_2CO$]. Thermal ellipsoids shown with 50% probability. Atoms generated by symmetry operations are shown at 50% visibility.

Table S2: Fractional Atomic Coordinates (×10^4) and Equivalent Isotropic Displacement Parameters (Å^2×10^3) for [$(HO_3SCH_2)_2CO$]. U_{eq} is defined as 1/3 of the trace of the orthogonalised U_{IJ} tensor.

Atom	*x*	*y*	*z*	U(eq)
S1	3741.4(7)	3569.2(13)	3933.8(3)	11.90(19)
O1	5000	1466(6)	2500	34.7(9)
O11	5078(2)	1827(4)	4248.0(12)	21.8(4)
O12	2637(2)	1710(4)	3590.5(12)	19.8(4)
O13	3347(2)	5435(4)	4567.8(11)	19.7(4)
C1	5000	3974(8)	2500	18.7(7)
C2	4349(3)	5678(5)	3156.1(15)	14.3(5)

Table S3: Anisotropic Displacement Parameters (Å^2×10^3) for [$(HO_3SCH_2)_2CO$]. The anisotropic displacement factor exponent takes the form: $-2\pi^2[h^2a^{*2}U_{11}+2hka^*b^*U_{12}+\ldots]$.

Atom	U_{11}	U_{22}	U_{33}	U_{23}	U_{13}	U_{12}
S1	17.0(3)	9.8(3)	8.9(3)	-0.6(2)	1.1(2)	-0.5(2)
O1	70(2)	11.8(13)	26.7(16)	0	27.7(16)	0
O11	26.8(10)	18.2(10)	19.1(9)	2.4(8)	-4.0(8)	1.9(8)
O12	21.8(10)	17.4(9)	20.2(9)	-2.7(7)	1.8(7)	-5.1(8)
O13	29.7(10)	17.0(9)	13.6(8)	-4.6(7)	7.6(7)	-0.1(8)
C1	30(2)	13.0(16)	14.5(16)	0	7.0(14)	0
C2	20.9(12)	10.0(10)	12.3(10)	1.4(9)	3.6(9)	0.5(9)

Table S4: Bond Lengths for [$(HO_3SCH_2)_2CO$].

Atom	Atom	Length/pm	Atom	Atom	Length/pm
S1	O11	153.3(2)	O1	C1	120.1(5)
S1	O12	142.5(2)	C1	C2[1]	152.3(3)
S1	O13	144.7(2)	C1	C2	152.3(3)
S1	C2	176.4(2)			

[1]1-X,+Y,1/2-Z

Table S5: Bond Angles for [$(HO_3SCH_2)_2CO$].

Atom	Atom	Atom	Angle/°	Atom	Atom	Atom	Angle/°
O11	S1	C2	104.8(1)	O13	S1	C2	106.8(1)
O12	S1	O11	108.1(1)	O1	C1	C2	122.4(2)
O12	S1	O13	116.9(1)	O1	C1	C2[1]	122.4(2)
O12	S1	C2	109.5(1)	C2[1]	C1	C2	115.2(3)
O13	S1	O11	110.1(1)	C1	C2	S1	112.5(2)

[1]1-X,+Y,1/2-Z

Table S6: Torsion Angles for [$(HO_3SCH_2)_2CO$].

A	B	C	D	Angle/°	A	B	C	D	Angle/°
O1	C1	C2	S1	4.4(2)	O13	S1	C2	C1	172.0(2)
O11	S1	C2	C1	55.3(2)	C2[1]	C1	C2	S1	-175.6(2)
O12	S1	C2	C1	-60.5(2)					

[1]1-X,+Y,1/2-Z

Table S7: Hydrogen Atom Coordinates (Å×10^4) and Isotropic Displacement Parameters (Å^2×10^3) for [$(HO_3SCH_2)_2CO$].

Atom	*x*	*y*	*z*	U(eq)
H11	5561.27	2685.72	4628.94	33
H2A	3516.57	6782.86	2900.61	17
H2B	5093.99	7001.43	3401.16	17

$[NH_4]_2[(O_3SCH_2)_2CO]$ (2)

Table S8: Crystallographic data of $[NH_4]_2[(O_3SCH_2)_2CO]$.

Empirical formula	$C_3H_{12}N_2O_7S_2$
Formula weight	252.27 g/mol
Temperature	260.00 K
Crystal system	Orthorhombic
Space group	Pbcn (No. 60)
Unit cell dimensions	a = 747.38(3) pm<br>b = 763.44(4) pm<br>c = 1713.59(9) pm<br>α = 90°<br>β = 90°<br>γ = 90°
Volume	977.74(8) $Å^3$
Z	4
ρ_{calc}	1.714 g/cm^3
μ	0.562 mm^{-1}
F(000)	528
Radiation	MoK_α (λ = 0.71073 nm)
Crystal size	0.3 x 0.3 x 0.3 mm^3
2Θ range for data collection	4.754 to 61.02
Index ranges	$-10 \leq h \leq 10$, $-10 \leq k \leq 10$, $-24 \leq l \leq 24$
Reflections collected	33557
Independent reflections	1495 [R_{int} = 0.0502, R_σ = 0.0199]
Completeness	99.9%
Absorption correction	multiscan
Min. and max. transmission	0.707 / 0.747
Data/restraints/parameters	1495/0/89
Goodness-of-fit on F^2	1.105
Final R indexes [I≥2σ (I)]	R_1 = 0.0282, wR_2 = 0.0799
Final R indexes [all data]	R_1 = 0.0316, wR_2 = 0.0824
Largest diff. peak/hole	0.28/–0.37 e · $Å^{-3}$
CCDC-No.	2533289

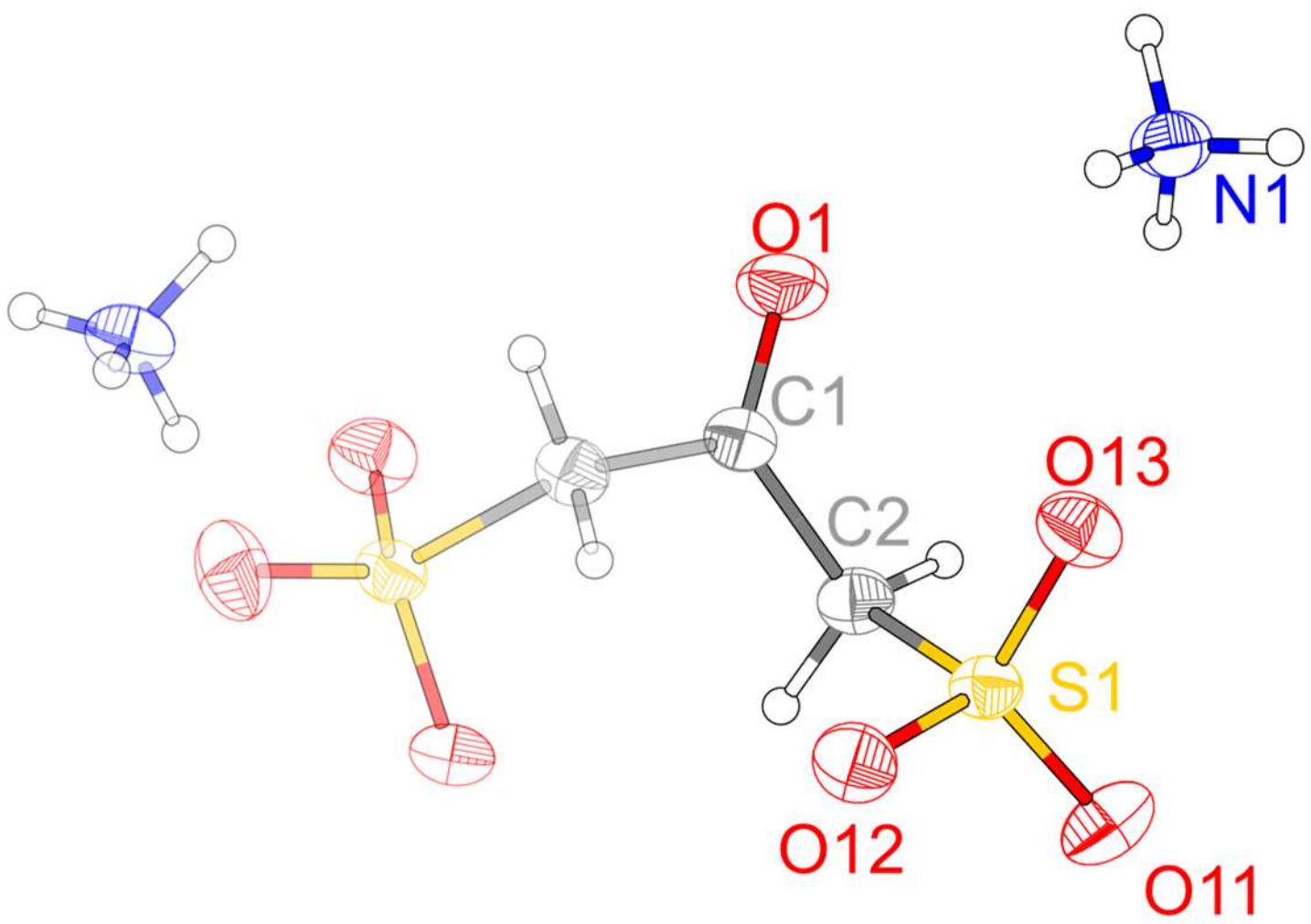

Figure S2: Thermal ellipsoid plot of the asymmetric unit of $[NH_4]_2[(O_3SCH_2)_2CO]$. Thermal ellipsoids shown with 50% probability. Atoms generated by symmetry operations are shown at 50% visibility.

Table S9: Fractional Atomic Coordinates (×10[4]) and Equivalent Isotropic Displacement Parameters (Å^2×10^3) for $[NH_4]_2[(O_3SCH_2)_2CO]$. U_{eq} is defined as 1/3 of the trace of the orthogonalised U_{IJ} tensor.

Atom	*x*	*y*	*z*	U(eq)
S1	5515.7(4)	3744.3(4)	3814.2(2)	26.61(11)
O1	5000	185.6(17)	2500	42.8(3)
O11	7031.8(13)	4442.7(14)	4234.2(6)	43.2(3)
O12	4254.6(14)	5083.8(12)	3561.2(6)	39.0(2)
O13	4605.0(13)	2327.0(13)	4218.9(6)	36.2(2)
C1	5000	1771(2)	2500	26.4(3)
C2	6403.1(15)	2775.7(15)	2946.4(7)	28.0(2)
N1	821.6(17)	3486.9(16)	4244.4(8)	

Table S10: Anisotropic Displacement Parameters (Å^2×10^3) for $[NH_4]_2[(O_3SCH_2)_2CO]$. The anisotropic displacement factor exponent takes the form: $-2\pi^2[h^2a^{*2}U_{11}+2hka^*b^*U_{12}+\ldots]$.

Atom	U_{11}	U_{22}	U_{33}	U_{23}	U_{13}	U_{12}
S1	27.24(16)	22.19(17)	30.40(17)	-1.55(9)	-0.23(9)	-0.23(9)
O1	47.5(7)	22.0(6)	59.0(9)	0	-14.5(7)	0
O11	35.4(5)	50.3(6)	43.9(5)	-14.2(5)	-4.7(4)	-8.2(4)
O12	47.6(5)	26.1(4)	43.1(5)	-3.9(4)	-3.6(4)	12.5(4)
O13	39.6(5)	31.1(5)	38.0(5)	4.2(4)	8.1(4)	-2.3(4)
C1	27.6(7)	22.2(7)	29.5(7)	0	1.5(6)	0
C2	25.0(5)	26.0(5)	32.9(5)	-1.8(4)	1.1(4)	-1.2(4)
N1	33.0(5)	26.4(5)	43.1(6)	-1.2(5)	7.8(5)	2.5(4)

Table S11: Bond Lengths [in pm] for $[NH_4]_2[(O_3SCH_2)_2CO]$.

Atom	Atom	Length/pm	Atom	Atom	Length/pm
S1	O11	144.4(1)	O1	C1	121.0(2)
S1	O12	145.7(1)	C1	C2	150.8(1)
S1	O13	145.43(9)	C1	C2[1]	150.8(1)
S1	C2	178.8(1)			

[1]1-X,+Y,1/2-Z.

Table S12: Bond Angles for $[NH_4]_2[(O_3SCH_2)_2CO]$.

Atom	Atom	Atom	Angle/°	Atom	Atom	Atom	Angle/°
O11	S1	O12	113.38(6)	O13	S1	C2	105.21(6)
O11	S1	O13	113.85(6)	O1	C1	C2	120.58(7)
O11	S1	C2	106.02(6)	O1	C1	C2[1]	120.58(7)
O12	S1	C2	106.42(6)	C2	C1	C2[1]	118.84(14)
O13	S1	O12	111.19(6)	C1	C2	S1	111.98(7)

[1]1-X,+Y,1/2-Z.

Table S13: (Reduced) Torsion Angles for $[NH_4]_2[(O_3SCH_2)_2CO]$. Angles including Rubidium atoms were omitted for clarity.

A	B	C	D	Angle/°	A	B	C	D	Angle/°
O1	C1	C2	S1	-106.23(6)	O13	S1	C2	C1	51.23(10)
O11	S1	C2	C1	172.16(9)	C2[1]	C1	C2	S1	73.77(6)
O12	S1	C2	C1	-66.85(10)					

[1]1-X,+Y,1/2-Z.

Table S14: Hydrogen Atom Coordinates (Å×10^4) and Isotropic Displacement Parameters (Å^2×10^3) for $[NH_4]_2[(O_3SCH_2)_2CO]$.

Atom	*x*	*y*	*z*	U(eq)
H2A	7300(20)	1950(20)	3121(10)	41(4)
H2B	6860(20)	3780(20)	2648(9)	38(4)
H1A	780(30)	3340(30)	4753(16)	77(7)
H1B	1640(40)	4090(30)	4126(15)	91(9)
H1C	780(30)	2400(40)	3976(13)	74(7)
H1D	-230(40)	4030(30)	4105(17)	96(9)

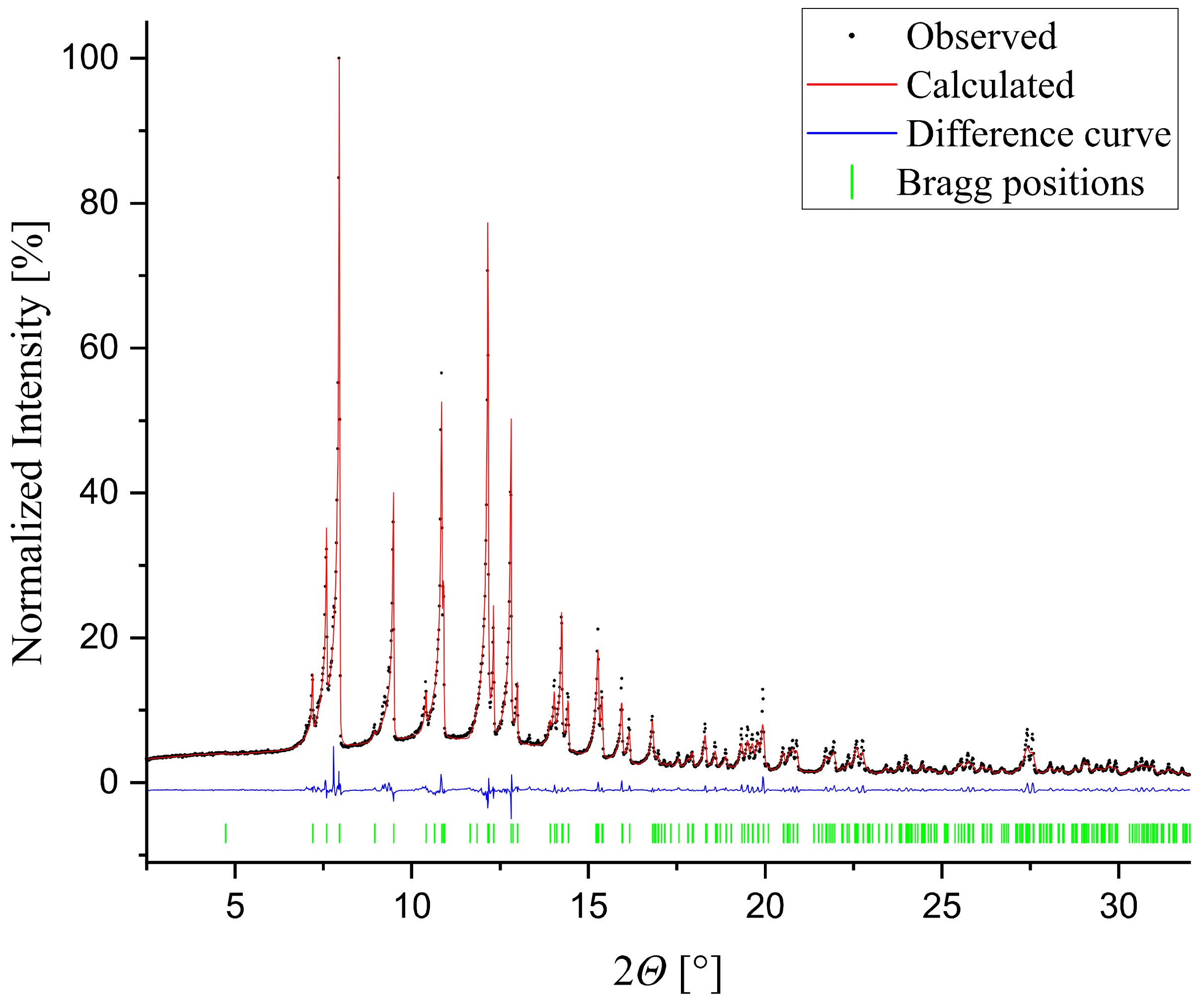

Figure S 3: Powder X-Ray Diffraction measurement of $[NH_4]_2[(O_3SCH_2)_2CO]$. Observed reflexes, calculated pattern and the Bragg positions of **2** are shown as black dots, a red curve and green stripes, respectively. Major deviations indicated by the difference curve are due to incomplete peak shape fitting.

The final cell parameters after *Rietveld* refinement are a = 750.2(2) pm, b = 764.8(2) pm, c = 1715.8(4) pm, V = 984.4(5) Å^3 with *Rwp* = 5.734, *Rexp* = 1.385, *Rp* = 3.948 and *GooF* = 4.138.

$K_2[(O_3SCH_2)_2CO]$ (3)

Table S15: Crystallographic data of $K_2[(O_3SCH_2)_2CO]$.

Empirical formula	$C_3H_4K_2O_7S_2$
Formula weight	294.38 g/mol
Temperature	100.00 K
Crystal system	Orthorhombic
Space group	Pbcn (No. 60)
Unit cell dimensions	a = 730.44(4) pm<br>b = 739.99(4) pm<br>c = 1637.8(1) pm<br>α = 90°<br>β = 90°<br>γ = 90°
Volume	885.26(9) Å^3
Z	4
ρ_{calc}	2.209 g/cm^3
μ	1.549 mm^{-1}
F(000)	592
Radiation	MoK_α (λ = 0.71073 nm)
Crystal size	0.06 x 0.06 x 0.03 mm^3
2Θ range for data collection	7.476 to 57.389
Index ranges	$-9 \leq h \leq 9$, $-9 \leq k \leq 9$, $-22 \leq l \leq 21$
Reflections collected	8600
Independent reflections	1121 [R_{int} = 0.0319, R_σ = 0.0285]
Completeness	98.4%
Absorption correction	multiscan
Min. and max. transmission	0.668 / 0.747
Data/restraints/parameters	1121/0/65
Goodness-of-fit on F^2	1.118
Final R indexes [I≥2σ (I)]	R_1 = 0.0285, wR_2 = 0.0605
Final R indexes [all data]	R_1 = 0.0319, wR_2 = 0.0640
Largest diff. peak/hole	0.48/–0.45 e · Å^{-3}
CCDC-No.	2517620

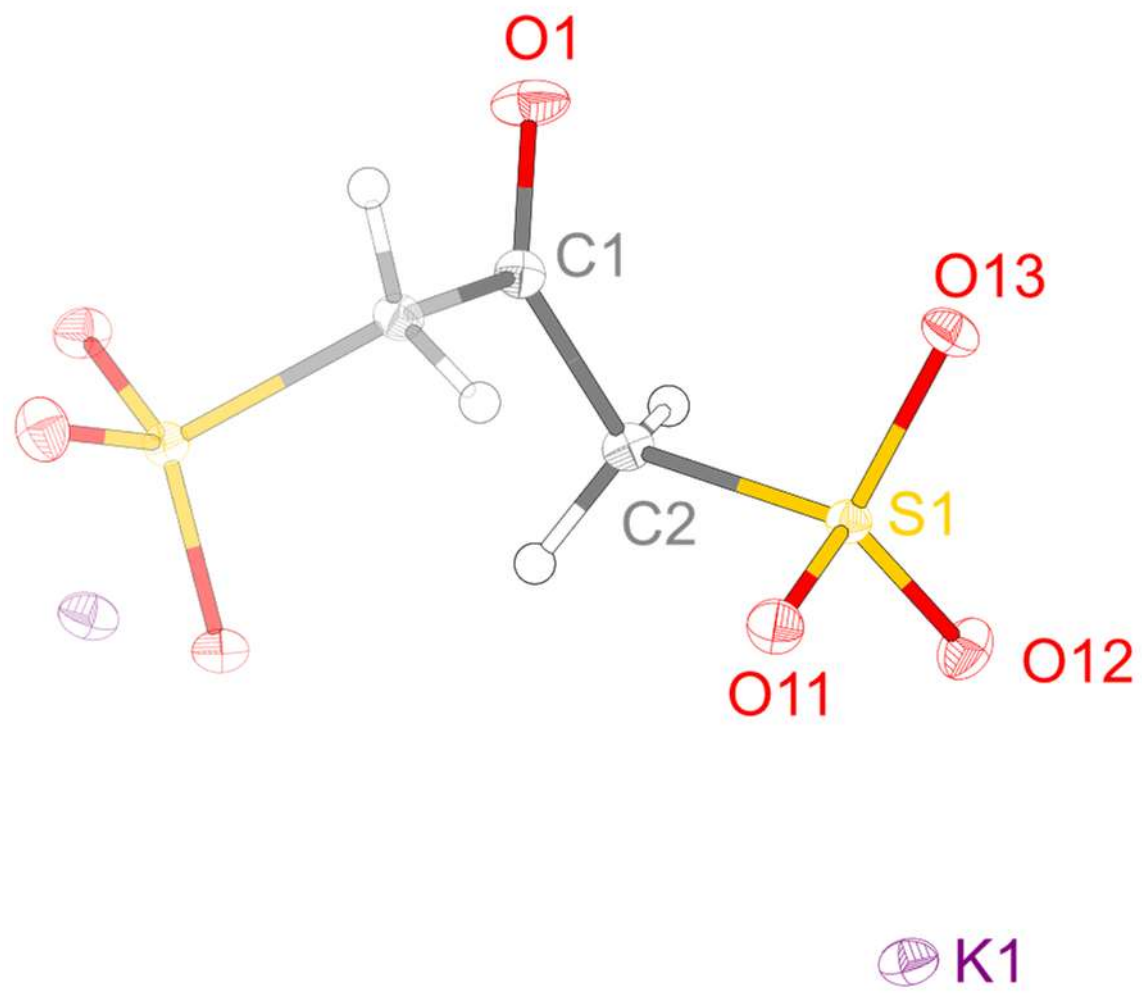

Figure S4: Thermal ellipsoid plot of the asymmetric unit of $K_2[(O_3SCH_2)_2CO]$.Thermal ellipsoids shown with 50% probability. Atoms generated by symmetry operations are shown at 50% visibility.

Table S16: Fractional Atomic Coordinates (×10^4) and Equivalent Isotropic Displacement Parameters (Å^2×10^3) for $K_2[(O_3SCH_2)_2CO]$. U_{eq} is defined as 1/3 of the trace of the orthogonalised U_{IJ} tensor.

Atom	*x*	*y*	*z*	U(eq)
K1	3940.3(6)	1362.6(5)	4264.1(3)	12.65(12)
S1	5629.2(6)	6238.7(6)	3869.3(3)	9.12(12)
O1	5000	9892(3)	2500	18.3(4)
O11	4448.2(19)	4736.4(18)	3630.9(9)	13.7(3)
O12	7240(2)	5696(2)	4326.2(8)	15.1(3)
O13	4591.4(19)	7684.1(18)	4260.8(8)	12.8(3)
C1	5000	8249(4)	2500	10.0(5)
C2	6473(3)	7209(3)	2938.7(11)	10.4(3)

Table S17: Anisotropic Displacement Parameters (Å^2×10^3) for $K_2[(O_3SCH_2)_2CO]$. The anisotropic displacement factor exponent takes the form: $-2\pi^2[h^2a^{*2}U_{11}+2hka^*b^*U_{12}+\dots]$.

Atom	U_{11}	U_{22}	U_{33}	U_{23}	U_{13}	U_{12}
K1	11.3(2)	10.2(2)	16.5(2)	0.75(15)	-2.84(14)	0.19(14)
S1	8.8(2)	8.9(2)	9.7(2)	-0.21(15)	-0.20(15)	0.32(15)
O1	16.6(9)	9.0(9)	29.3(11)	0	-7.4(9)	0
O11	15.9(7)	10.5(6)	14.8(7)	-0.2(5)	-0.5(5)	-3.7(5)
O12	12.1(7)	19.3(7)	13.9(6)	2.9(5)	-2.0(5)	1.1(6)
O13	12.3(7)	11.0(6)	15.0(6)	-2.5(5)	3.6(5)	0.8(5)
C1	6.9(11)	11.6(12)	11.7(11)	0	1.1(9)	0
C2	8.7(8)	10.5(8)	12.0(8)	1.0(7)	0.0(6)	-0.4(6)

Table S18: Bond Lengths and Interatomic Distances [in pm] for $K_2[(O_3SCH_2)_2CO]$.

Atom	Atom	Length/pm	Atom	Atom	Length/pm
K1	K1^1	350.35(8)	K1	O13^4	276.3(1)
K1	S1^2	354.92(6)	K1	O13^2	273.6(2)
K1	S1^3	340.11(6)	S1	O11	146.0(1)
K1	O1^4	318.29(8)	S1	O12	145.1(1)
K1	O11^3	294.10(2)	S1	O13	145.9(1)
K1	O11	272.9(1)	S1	C2	179.4(2)
K1	O12^5	303.3(1)	O1	C1	121.5(3)
K1	O12^6	283.5(2)	C1	C2^7	150.6(2)
K1	O12^2	328-8(2)	C1	C2	150.6(2)
K1	O13^3	275.9(2)			

^{1}1-X,-Y,1-Z; ^{2}1-X,1-Y,1-Z; ^{3}1/2-X,-1/2+Y,+Z; ^{4}+X,-1+Y,+Z; ^{5}-1/2+X,1/2-Y,1-Z; ^{6}3/2-X,-1/2+Y,+Z; ^{7}1-X,+Y,1/2-Z

Table S19: (Reduced) Bond Angles for $K_2[(O_3SCH_2)_2CO]$. Angles including Potassium atoms were omitted for clarity.

Atom	Atom	Atom	Angle/°	Atom	Atom	Atom	Angle/°
O11	S1	C2	106.30(9)	O13	S1	C2	104.97(9)
O12	S1	O11	113.98(9)	O1	C1	C2^{12}	120.7(1)
O12	S1	O13	113.41(9)	O1	C1	C2	120.7(1)
O12	S1	C2	105.68(9)	C2^{12}	C1	C2	118.5(2)
O13	S1	O11	111.62(8)	C1	C2	S1	111.4(1)

[12]1-X,+Y,1/2-Z

Table S20: (Reduced) Torsion Angles for $K_2[(O_3SCH_2)_2CO]$. Angles including Potassium atoms were omitted for clarity.

A	B	C	D	Angle/°	A	B	C	D	Angle/°
O1	C1	C2	S1	105.51(1)	O13	S1	C2	C1	-45.5(2)
O11	S1	C2	C1	72.9(2)	C2[7]	C1	C2	S1	-74.5(1)
O12	S1	C2	C1	-165.7(1)					

[7]1-X,+Y,1/2-Z

Table S21: Hydrogen Atom Coordinates (Å×10^4) and Isotropic Displacement Parameters (Å^2×10^3) for $K_2[(O_3SCH_2)_2CO]$.

Atom	*x*	*y*	*z*	U(eq)
H2A	7507.54	8028.35	3063.9	13
H2B	6934.89	6235.81	2578.87	13

$Rb_2[(O_3SCH_2)_2CO]$ (4)

Table S22: Crystallographic data of $Rb_2[(O_3SCH_2)_2CO]$.

Empirical formula	$C_3H_4Rb_2O_7S_2$
Formula weight	387.12 g/mol
Temperature	100.00 K
Crystal system	Monoclinic
Space group	$P2_1/c$ (No. 14)
Unit cell dimensions	a = 1701.40(9) pm<br>b = 752.06(3) pm<br>c = 757.14(4) pm<br>α = 90°<br>β = 94.562(2)°<br>γ = 90°
Volume	965.73(8) Å^3
Z	4
ρ_{calc}	2.663 g/cm^3
μ	10.574 mm^{-1}
F(000)	736
Radiation	MoK_α (λ = 0.71073 nm)
Crystal size	0.32 x 0.26 x 0.06 mm^3
2Θ range for data collection	4.804 to 66.266
Index ranges	$-26 \leq h \leq 26$, $-11 \leq k \leq 11$, $-11 \leq l \leq 11$
Reflections collected	36192
Independent reflections	3610 [R_{int} = 0.0450, R_σ = 0.0209]
Completeness	99.6%
Absorption correction	multiscan
Min. and max. transmission	0.240 / 0.747
Data/restraints/parameters	3610/0/139
Goodness-of-fit on F^2	1.091
Final R indexes [$I \geq 2\sigma$ (I)]	R_1 = 0.0255, wR_2 = 0.0704
Final R indexes [all data]	R_1 = 0.0290, wR_2 = 0.0756
Largest diff. peak/hole	0.95/–1.00 e · Å^{-3}
CCDC-No.	2517626

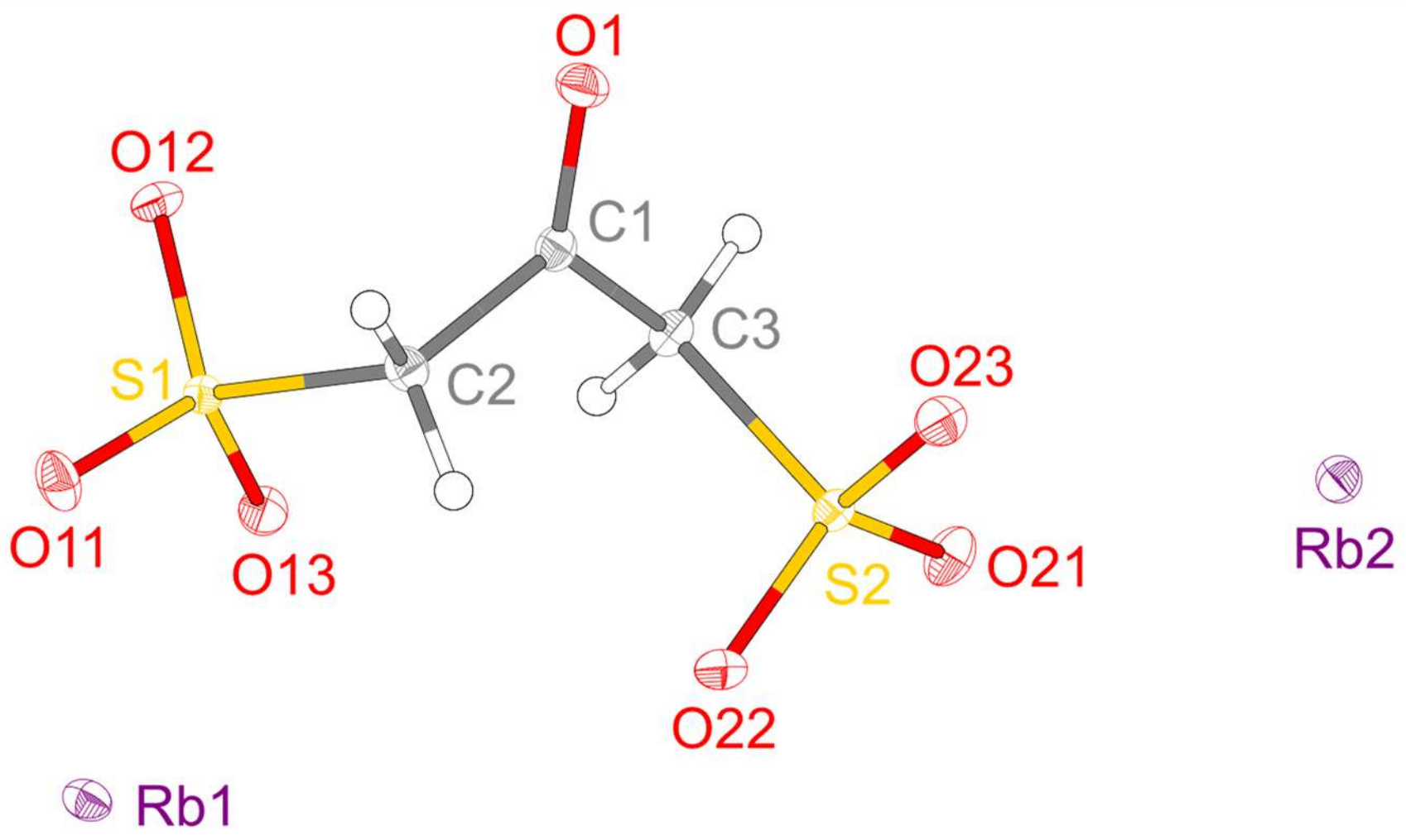

Figure S5: Thermal ellipsoid plot of the asymmetric unit of $Rb_2[(O_3SCH_2)_2CO]$. Thermal ellipsoids shown with 50% probability.

Table S23: Fractional Atomic Coordinates (×10^4) and Equivalent Isotropic Displacement Parameters (Å^2×10^3) for $Rb_2[(O_3SCH_2)_2CO]$. U_{eq} is defined as 1/3 of the trace of the orthogonalised U_{IJ} tensor.

**Atom**	***x***	***y***	***z***	**U(eq)**
Rb1	758.9(2)	6064.2(3)	1517.1(3)	10.71(6)
Rb2	5824.1(2)	5583.8(3)	8067.5(3)	11.36(6)
S1	1124.7(3)	4313.8(6)	6478.0(7)	8.26(9)
S2	3758.1(3)	5251.3(7)	6594.5(7)	9.38(9)
O1	2486.9(10)	5052(2)	10172(2)	14.2(3)
O11	666.0(10)	2777(2)	5877(2)	14.6(3)
O12	759.0(10)	5359(2)	7816(2)	11.7(3)
O13	1369.6(10)	5433(2)	5045(2)	12.3(3)
O21	4175.6(10)	6726(2)	5855(2)	15.2(3)
O22	3456.7(11)	3972(2)	5267(2)	14.6(3)
O23	4207.2(10)	4398(2)	8085(2)	13.1(3)
C1	2475.7(12)	4911(3)	8570(3)	9.8(3)
C2	2011.8(12)	3445(3)	7601(3)	10.0(3)
C3	2921.2(13)	6227(3)	7514(3)	10.4(3)

Table S24: Anisotropic Displacement Parameters (Å^2×10^3) for $Rb_2[(O_3SCH_2)_2CO]$. The anisotropic displacement factor exponent takes the form: $-2\pi^2[h^2a^{*2}U_{11}+2hka^*b^*U_{12}+\ldots]$.

**Atom**	**U_{11}**	**U_{22}**	**U_{33}**	**U_{23}**	**U_{13}**	**U_{12}**
Rb1	13.67(10)	9.69(9)	8.75(10)	-0.66(6)	0.78(7)	-2.21(6)
Rb2	11.5(1)	11.98(10)	10.66(10)	1.08(6)	1.22(7)	-1.88(6)
S1	8.2(2)	8.33(18)	8.3(2)	-0.38(14)	0.61(15)	0.05(15)
S2	8.9(2)	9.87(19)	9.6(2)	0.75(15)	2.12(16)	0.02(15)
O1	15.9(8)	17.2(7)	9.5(7)	-1.3(5)	1.3(6)	-3.2(6)
O11	12.6(7)	10.9(6)	19.6(8)	-3.0(6)	-3.1(6)	-2.3(5)
O12	12.7(7)	12.7(6)	10.1(7)	-0.8(5)	3.9(5)	2.3(5)
O13	12.3(7)	15.3(7)	9.2(7)	3.7(5)	0.1(5)	0.6(5)
O21	13.4(7)	14.7(7)	18.1(8)	4.1(6)	5.4(6)	-1.8(6)
O22	14.7(7)	18.1(7)	11.4(7)	-4.7(5)	3.4(6)	-1.0(6)
O23	13.2(7)	14.4(7)	11.5(7)	2.3(5)	0.5(5)	2.8(5)
C1	8.3(8)	10.0(8)	11.0(8)	0.2(6)	0.3(6)	1.8(6)
C2	10.8(8)	7.9(7)	11.0(8)	-0.6(6)	0.0(6)	-0.3(6)
C3	9.7(8)	9.7(7)	12.2(8)	0.6(6)	2.8(7)	0.4(6)

Table S25: Bond Lengths and Interatomic Distances [in pm] for $Rb_2[(O_3SCH_2)_2CO]$.

Atom	Atom	Length/pm	Atom	Atom	Length/pm
Rb1	Rb1[1]	368.29(4)	Rb2	O22[8]	291.1(2)
Rb1	S1[2]	366.46(6)	Rb2	O22[7]	305.5(2)
Rb1	S1[3]	353.18(5)	Rb2	O23	289.3(1)
Rb1	O1[4]	327.7(2)	Rb2	O23[9]	291.8(2)
Rb1	O11[5]	293.1(1)	Rb2	O23[7]	299.8(2)
Rb1	O11[6]	318.2(1)	Rb2	C1[9]	371.7(2)
Rb1	O11[2]	336.0(2)	S1	O11	144.8(2)
Rb1	O12[3]	286.4(2)	S1	O12	146.0(2)
Rb1	O12[4]	285.2(2)	S1	O13	146.0(2)
Rb1	O12[2]	287.7(2)	S1	C2	179.6(2)
Rb1	O13	282.8(2)	S2	O21	145.3(2)
Rb1	O13[3]	307.3(2)	S2	O22	145.5(2)
Rb2	S2[7]	358.66(6)	S2	O23	146.0(2)
Rb2	S2[8]	370.93(6)	S2	C3	179.1(2)
Rb2	O1[9]	310.7(2)	O1	C1	121.7(3)
Rb2	O21[10]	301.4(2)	C1	C2	151.2(3)
Rb2	O21[8]	344.1(2)	C1	C3	151.3(3)
Rb2	O21	326.6(2)			

[1]-X,1-Y,-Z; [2]-X,1-Y,1-Z; [3]+X,3/2-Y,-1/2+Z; [4]+X,+Y,-1+Z; [5]+X,1/2-Y,-1/2+Z; [6]-X,1/2+Y,1/2-Z; [7]1-X,1/2+Y,3/2-Z; [8]1-X,1-Y,1-Z; [9]1-X,1-Y,2-Z; [10]1-X,-1/2+Y,3/2-Z

Table S26: (Reduced) Bond Angles for $Rb_2[(O_3SCH_2)_2CO]$. Angles including Rubidium atoms were omitted for clarity.

Atom	Atom	Atom	Angle/°	Atom	Atom	Atom	Angle/°
O11	S1	O12	113.52(10)	O21	S2	C3	105.38(10)
O11	S1	O13	113.92(11)	O22	S2	O23	112.16(11)
O11	S1	C2	105.66(10)	O22	S2	C3	106.98(10)
O11	S1	O12	113.52(10)	O23	S2	C3	105.26(10)
O12	S1	C2	104.84(10)	O1	C1	C2	120.91(19)
O13	S1	O12	111.57(10)	O1	C1	C3	120.1(2)
O13	S1	C2	106.44(10)	C2	C1	C3	119.03(18)
O21	S2	O22	113.25(11)	C1	C2	S1	110.54(14)
O21	S2	O23	113.04(11)	C1	C3	S2	112.59(14)

Table S27: (Reduced) Torsion Angles for $Rb_2[(O_3SCH_2)_2CO]$. Angles including Rubidium atoms were omitted for clarity.

A	B	C	D	Angle/°	A	B	C	D	Angle/°
O1	C1	C2	S1	104.9(2)	O21	S2	C3	C1	-172.12(15)
O1	C1	C3	S2	110.6(2)	O22	S2	C3	C1	67.08(18)
O11	S1	C2	C1	-170.00(15)	O23	S2	C3	C1	-52.39(18)
O12	S1	C2	C1	-49.79(17)	C2	C1	C3	S2	-70.2(2)
O13	S1	C2	C1	68.55(16)	C3	C1	C2	S1	-74.3(2)

Table S28: Hydrogen Atom Coordinates (Å×10^4) and Isotropic Displacement Parameters (Å^2×10^3) for $Rb_2[(O_3SCH_2)_2CO]$.

Atom	*x*	*y*	*z*	U(eq)
H2A	1880(20)	2600(50)	8420(50)	12
H2B	2310(20)	2900(50)	6750(50)	12
H3A	3100(20)	7190(50)	8280(50)	12
H3B	2620(20)	6640(50)	6540(50)	12

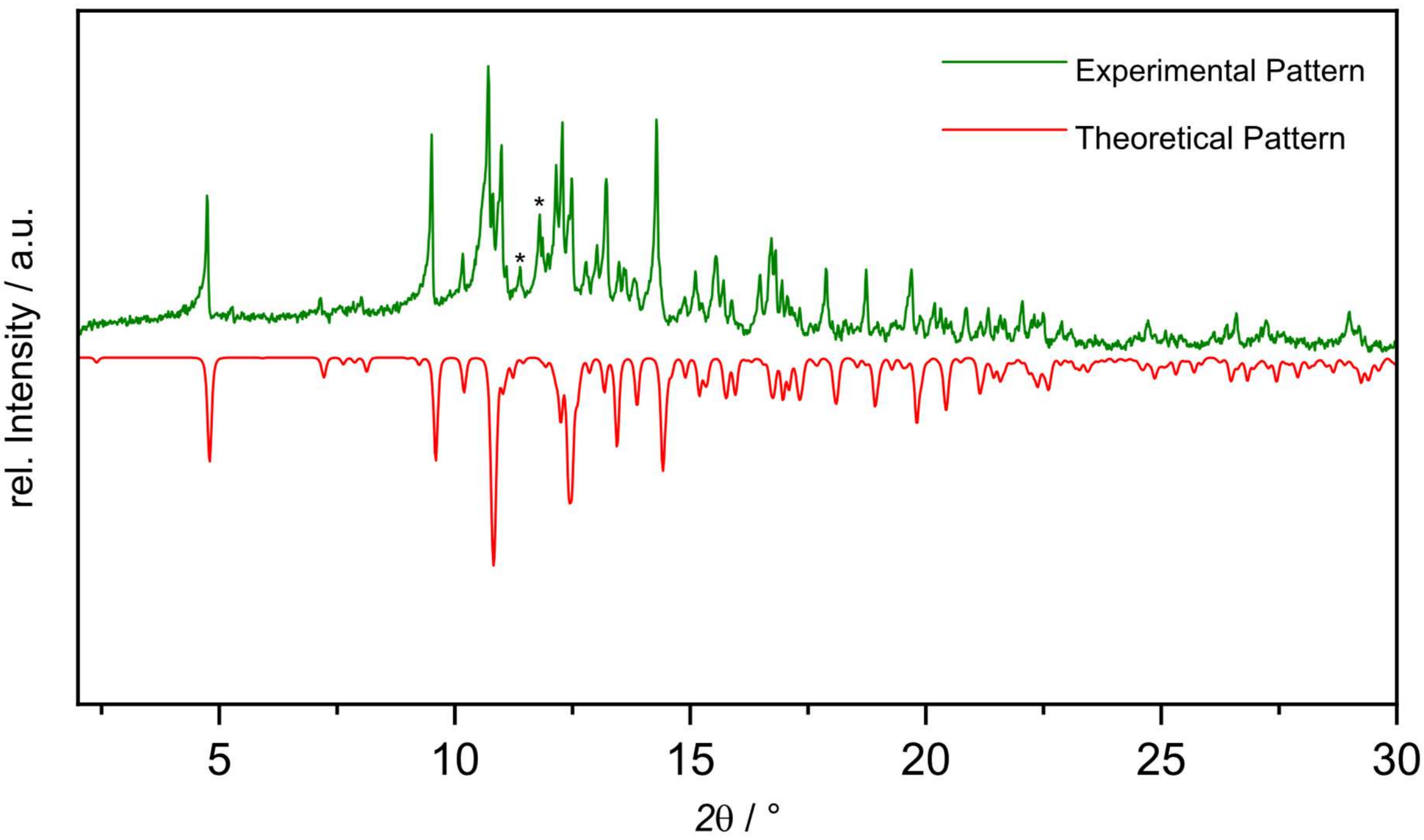

Figure S 6: Powder X-Ray Diffraction measurement of $Rb_2[(O_3SCH_2)_2CO]$. Observed pattern and calculated pattern of **4** are shown as dark green and red curves, respectively. Peaks of the unidentified phase are marked with an asterisk.

$[H_6C_5N]_3[(O_3SCH_2)_2CO][HSO]_4$ (5)

Table S29: Crystallographic data of $[H_6C_5N]_3[(O_3SCH_2)_2CO][HSO]_4$.

Empirical formula	$C_{18}H_{23}N_3O_{11}S_3$
Formula weight	553.57 g/mol
Temperature	100.00 K
Crystal system	Orthorhombic
Space group	$Pca2_1$ (No. 29)
Unit cell dimensions	a = 1909.4(3) pm<br>b = 1017.7(2) pm<br>c = 1167.9(2) pm<br>α = 90°<br>β = 90°<br>γ = 90°
Volume	2269.4(7) $Å^3$
Z	4
ρ_{calc}	1.620 g/cm^3
μ	0.394 mm^{-1}
F(000)	1152
Radiation	MoK_α (λ = 0.71073 nm)
Crystal size	0.09 x 0.07 x 0.01 mm^3
2Θ range for data collection	4.002 to 50.69
Index ranges	$-23 \leq h \leq 23$, $-12 \leq k \leq 12$, $-14 \leq l \leq 14$
Reflections collected	63289
Independent reflections	4156 [R_{int} = 0.1128, R_σ = 0.0380]
Completeness	99.9%
Absorption correction	multiscan
Min. and max. transmission	0.521 / 0.746
Data/restraints/parameters	4156/3/326
Goodness-of-fit on F^2	1.093
Final R indexes [I≥2σ (I)]	R_1 = 0.0700, wR_2 = 0.1517
Final R indexes [all data]	R_1 = 0.0892, wR_2 = 0.1653
Largest diff. peak/hole	0.60/–0.62 e · $Å^{-3}$
Flack parameter	0.3(2)
CCDC-No.	2526344

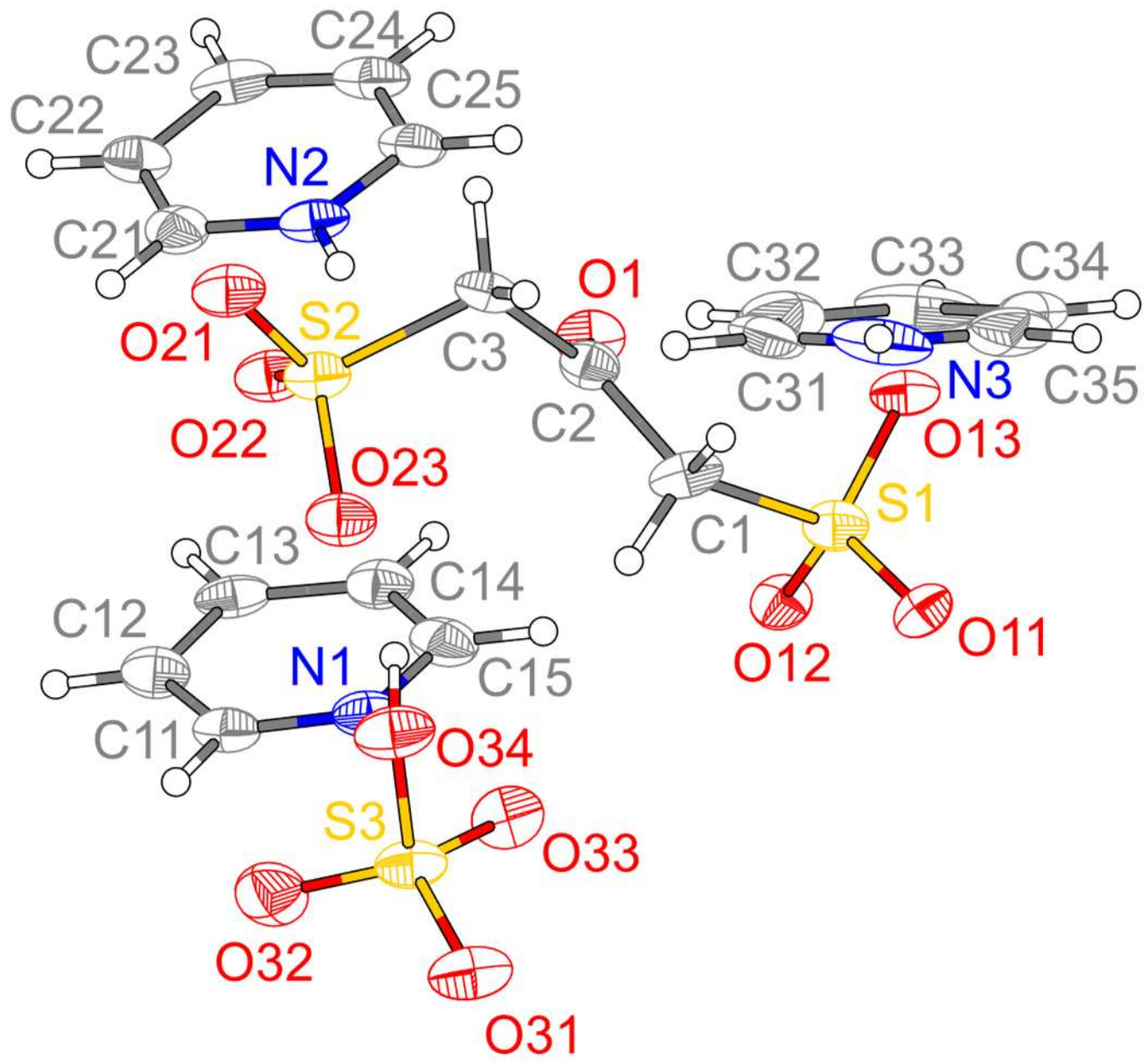

Figure S7: Thermal ellipsoid plot of the asymmetric unit of $[H_6C_5N]_3[(O_3SCH_2)_2CO][HSO]_4$. Thermal ellipsoids shown with 50% probability.

Table S30: Fractional Atomic Coordinates ($\times10^4$) and Equivalent Isotropic Displacement Parameters ($Å^2\times10^3$) for $[H_6C_5N]_3[(O_3SCH_2)_2CO][HSO]_4$. U_{eq} is defined as 1/3 of the trace of the orthogonalised U_{IJ} tensor.

Atom	*x*	*y*	*z*	U(eq)
S1	6177.4(14)	3768(2)	4852(2)	28.3(5)
S2	3676.8(14)	2937(2)	6537(2)	28.9(6)
O1	4974(4)	5048(7)	6103(6)	32.5(15)
O11	6620(3)	2715(6)	4446(6)	28.4(16)
O12	5869(3)	4557(6)	3948(5)	28.8(15)
O13	6526(4)	4572(6)	5719(6)	29.8(15)
O21	3275(4)	2387(7)	7470(6)	34.5(17)
O22	3415(4)	4199(6)	6133(6)	33.2(17)
O23	3789(4)	2032(6)	5588(6)	32.7(17)
C1	5474(6)	2976(9)	5582(8)	29(2)
C2	4997(5)	3880(9)	6258(8)	28(2)
C3	4527(5)	3229(10)	7125(8)	27(2)
S3	4105.1(14)	325(2)	3163(2)	32.1(6)
O31	4407(4)	-783(7)	2577(6)	43(2)
O32	3401(4)	662(7)	2827(7)	44(2)
O33	4560(4)	1463(6)	3171(6)	35.2(16)
O34	4067(4)	-164(7)	4462(6)	38.3(18)
N1	3822(4)	3769(8)	3339(7)	30(2)
C11	3127(5)	3777(9)	3230(8)	29(2)
C12	2755(6)	4917(9)	3367(8)	32(2)
C13	3121(6)	6053(10)	3630(8)	31(2)
C14	3850(5)	6022(10)	3714(8)	30(2)
C15	4187(6)	4856(11)	3584(8)	33(2)
N2	3583(5)	6814(8)	6603(7)	27.1(18)
C21	2899(5)	7099(10)	6462(8)	32(2)
C22	2678(6)	8353(10)	6665(8)	33(2)
C23	3144(6)	9306(9)	6995(8)	33(3)
C24	3836(6)	8964(10)	7111(8)	36(3)
C25	4061(6)	7711(11)	6933(8)	33(2)

N3	6351(8)	7138(10)	5323(8)	49(3)
C31	5765(8)	7784(15)	5180(9)	52(4)
C32	5803(8)	9087(15)	4904(11)	58(4)
C33	6464(10)	9655(11)	4790(9)	63(5)
C34	7046(7)	8897(17)	4958(11)	61(4)
C35	6963(8)	7626(13)	5218(10)	48(3)

Table S31: Anisotropic Displacement Parameters (Å^2×10^3) for $[H_6C_5N]_3[(O_3SCH_2)_2CO][HSO]_4$. The anisotropic displacement factor exponent takes the form: $-2\pi^2[h^2a^{*2}U_{11}+2hka^*b^*U_{12}+\ldots]$.

Atom	U_{11}	U_{22}	U_{33}	U_{23}	U_{13}	U_{12}
S1	40.7(14)	22.8(11)	21.3(11)	3.0(11)	0.3(11)	-1.3(12)
S2	41.3(14)	24.3(12)	20.9(11)	-0.6(11)	1.2(11)	-0.4(11)
O1	44(4)	24(3)	30(4)	1(3)	0(3)	2(3)
O11	32(4)	25(4)	28(3)	-2(3)	8(3)	3(3)
O12	35(4)	32(4)	20(3)	10(3)	1(3)	3(3)
O13	45(4)	23(3)	21(3)	-4(3)	0(3)	2(3)
O21	50(5)	36(4)	17(3)	9(3)	2(3)	-1(3)
O22	51(5)	23(4)	26(4)	-2(3)	4(3)	-3(3)
O23	46(4)	24(4)	28(4)	-2(3)	0(3)	-2(3)
C1	51(6)	16(4)	19(5)	3(4)	7(4)	3(4)
C2	33(5)	29(5)	20(5)	2(4)	-3(4)	-1(5)
C3	39(6)	25(5)	18(5)	-4(4)	-7(4)	0(4)
S3	48.4(16)	24.1(12)	23.8(12)	-4.4(11)	0.8(12)	2.6(11)
O31	66(5)	37(4)	28(4)	-5(4)	3(4)	7(4)
O32	49(5)	34(4)	48(5)	-13(4)	-4(4)	2(4)
O33	49(4)	22(3)	35(4)	4(3)	3(4)	3(3)
O34	61(5)	29(4)	25(4)	1(3)	5(3)	-1(4)
N1	45(5)	28(5)	16(4)	3(4)	-2(4)	5(4)
C11	43(6)	25(5)	18(4)	-1(4)	0(5)	-4(4)
C12	44(6)	31(5)	20(5)	5(4)	-2(4)	0(4)
C13	54(7)	24(5)	14(4)	2(4)	2(4)	7(5)
C14	43(6)	27(5)	19(5)	4(4)	2(4)	-5(5)
C15	44(6)	38(6)	18(5)	3(4)	-4(4)	-6(5)
N2	47(5)	18(4)	16(4)	0(3)	4(4)	-1(4)
C21	39(6)	39(6)	17(5)	3(5)	3(4)	-9(5)
C22	44(6)	35(5)	19(5)	-1(4)	-4(4)	2(5)
C23	67(8)	15(5)	18(4)	1(4)	9(5)	0(5)
C24	57(7)	34(6)	17(5)	-4(4)	2(4)	-10(5)
C25	43(6)	37(6)	19(5)	-6(4)	-2(4)	-5(5)
N3	94(9)	32(6)	20(5)	-2(4)	-12(5)	-5(6)
C31	58(9)	76(10)	21(6)	-15(6)	0(5)	-38(8)
C32	66(9)	70(10)	40(7)	-24(7)	-13(7)	40(8)
C33	152(17)	20(6)	15(5)	5(5)	-8(8)	-16(7)
C34	50(7)	102(12)	30(6)	-33(8)	10(6)	-24(8)
C35	68(9)	40(7)	38(7)	-25(5)	-6(6)	23(7)

Table S32: Bond Lengths for $[H_6C_5N]_3[(O_3SCH_2)_2CO][HSO]_4$.

Atom	Atom	Length/pm	Atom	Atom	Length/pm
S1	O11	144.4(7)	C11	C12	136.9(2)
S1	O12	145.2(7)	C12	C13	138.6(2)
S1	O13	146.3(7)	C13	C14	139.6(2)
S1	C1	178.3(1)	C14	C15	135.8(2)
S2	O21	144.5(7)	N2	C21	134.9(2)
S2	O22	145.7(7)	N2	C25	134.6(2)
S2	O23	145.7(7)	C21	C22	136.5(2)
S2	C3	178.8(1)	C22	C23	137.1(2)
O1	C2	120.3(1)	C23	C24	137.4(2)
C1	C2	151.5(1)	C24	C25	136.2(2)
C2	C3	150.7(1)	N3	C31	130.8(2)
S3	O31	144.0(8)	N3	C35	127.4(2)
S3	O32	144.2(8)	C31	C32	137(2)
S3	O33	144.8(7)	C32	C33	139(2)
S3	O34	159.8(7)	C33	C34	137(2)
N1	C11	133.3(1)	C34	C35	134(2)
N1	C15	133.8(1)			

Table S33: Bond Angles for $[H_6C_5N]_3[(O_3SCH_2)_2CO][HSO]_4$.

Atom	Atom	Atom	Angle/°	Atom	Atom	Atom	Angle/°
O11	S1	O12	114.2(4)	O32	S3	O34	106.8(5)
O11	S1	O13	112.1(4)	O33	S3	O34	105.7(4)
O11	S1	C1	105.2(4)	C11	N1	C15	122.3(9)
O12	S1	O13	112.3(4)	N1	C11	C12	120.6(9)
O12	S1	C1	107.0(4)	C11	C12	C13	118.1(9)
O13	S1	C1	105.3(4)	C12	C13	C14	120.0(9)
O21	S2	O22	113.8(4)	C15	C14	C13	118.9(9)
O21	S2	O23	114.0(4)	N1	C15	C14	120.0(1)
O21	S2	C3	104.9(4)	C25	N2	C21	123.1(9)
O22	S2	O23	111.2(4)	N2	C21	C22	118.6(1)
O22	S2	C3	106.8(4)	C21	C22	C23	120.6(1)
O23	S2	C3	105.3(4)	C22	C23	C24	118.2(9)
C2	C1	S1	115.3(7)	C25	C24	C23	121.7(1)
O1	C2	C1	122.9(9)	N2	C25	C24	117.7(1)
O1	C2	C3	120.9(9)	C35	N3	C31	125.1(1)
C3	C2	C1	116.2(8)	N3	C31	C32	118.2(1)
C2	C3	S2	110.9(6)	C31	C32	C33	118.2(1)
O31	S3	O32	115.5(5)	C34	C33	C32	119.3(1)
O31	S3	O33	113.0(5)	C35	C34	C33	118.8(1)
O31	S3	O34	103.0(4)	N3	C35	C34	120.5(1)
O32	S3	O33	111.8(5)				

Table S34: Torsion Angles for $[H_6C_5N]_3[(O_3SCH_2)_2CO][HSO]_4$.

A	B	C	D	Angle/°	A	B	C	D	Angle/°
S1	C1	C2	O1	17.8(14)	C13	C14	C15	N1	2.3(14)
S1	C1	C2	C3	-164.1(7)	C15	N1	C11	C12	0.0(14)
O1	C2	C3	S2	82.1(11)	N2	C21	C22	C23	0.7(15)
O11	S1	C1	C2	170.4(7)	C21	N2	C25	C24	-1.0(14)
O12	S1	C1	C2	-67.9(8)	C21	C22	C23	C24	0.1(15)
O13	S1	C1	C2	51.8(8)	C22	C23	C24	C25	-1.4(15)
O21	S2	C3	C2	-176.8(7)	C23	C24	C25	N2	1.9(15)
O22	S2	C3	C2	-55.7(8)	C25	N2	C21	C22	-0.2(15)
O23	S2	C3	C2	62.6(8)	N3	C31	C32	C33	0.0(17)
C1	C2	C3	S2	-96.0(9)	C31	N3	C35	C34	1.0(18)
N1	C11	C12	C13	-0.5(14)	C31	C32	C33	C34	0.0(18)
C11	N1	C15	C14	-0.9(14)	C32	C33	C34	C35	0.5(18)
C11	C12	C13	C14	1.9(14)	C33	C34	C35	N3	-0.9(18)
C12	C13	C14	C15	-2.8(14)	C35	N3	C31	C32	-0.5(17)

Table S35: Hydrogen Atom Coordinates (Å×10^4) and Isotropic Displacement Parameters (Å^2×10^3) for $[H_6C_5N]_3[(O_3SCH_2)_2CO][HSO]_4$.

Atom	*x*	*y*	*z*	U(eq)
H1A	5187.53	2498.15	5011.92	34
H1B	5673.58	2316.7	6113.04	34
H3A	4485.28	3797.8	7808.79	33
H3B	4737.07	2383.87	7367.27	33
H34A	4065.38	490.03	4900.85	57
H1	3990(50)	2940(40)	3360(90)	20(30)
H11	2887.11	2983.75	3055.06	34
H12	2260.02	4928.16	3284.68	38
H13	2876.13	6852.91	3752.4	37
H14	4106.83	6803.88	3861	36
H15	4681.06	4812.05	3666.32	40
H2	3724.39	6004.67	6473.9	32
H21	2577.35	6440.6	6225.2	38
H22	2196.44	8566.86	6578.29	39
H23	2991.54	10179.38	7138.39	40
H24	4166.39	9618.72	7322.21	43
H25	4538.47	7477.79	7036.95	40
H3	6360(70)	6320(60)	5620(100)	60(40)
H31	5325.56	7359.49	5265.38	62
H32	5390.45	9592.49	4792.55	70
H33	6509.46	10557.73	4598.65	75
H34	7501.05	9265.92	4890.39	73
H35	7362.06	7082.95	5325.42	58

$Sr[(O_3SCH_2)_2CO](H_2O)_2$ (6)

Table S36: Crystallographic data of $Sr[(O_3SCH_2)_2CO](H_2O)_2$.

Empirical formula	$C_3H_8O_9S_2Sr$
Formula weight	339.83 g/mol
Temperature	100.00 K
Crystal system	Orthorhombic
Space group	$Pca2_1$ (No. 29)
Unit cell dimensions	a = 1087.7(2) pm<br>b = 725.3(1) pm<br>c = 1222.6(2) pm<br>α = 90°<br>β = 90°<br>γ = 90°
Volume	964.5(3) Å^3
Z	4
ρ_{calc}	2.340 g/cm^3
μ	6.053 mm^{-1}
F(000)	672
Radiation	MoK_α (λ = 0.71073 nm)
Crystal size	0.4 x 0.3 x 0.2 mm^3
2Θ range for data collection	5.618 to 61.002
Index ranges	$-15 \leq h \leq 15$, $-10 \leq k \leq 10$, $-17 \leq l \leq 17$
Reflections collected	27055
Independent reflections	2820 [R_{int} = 0.0540, R_σ = 0.0441]
Completeness	99.1%
Absorption correction	multiscan
Min. and max. transmission	0.461 / 0.746
Data/restraints/parameters	2820/1/142
Goodness-of-fit on F^2	1.068
Final R indexes [$I \geq 2\sigma$ (I)]	R_1 = 0.0197, wR_2 = 0.0438
Final R indexes [all data]	R_1 = 0.0211, wR_2 = 0.0449
Largest diff. peak/hole	0.49/–0.33 e · Å^{-3}
Flack parameter	0.035(5)
CCDC-No.	2533290

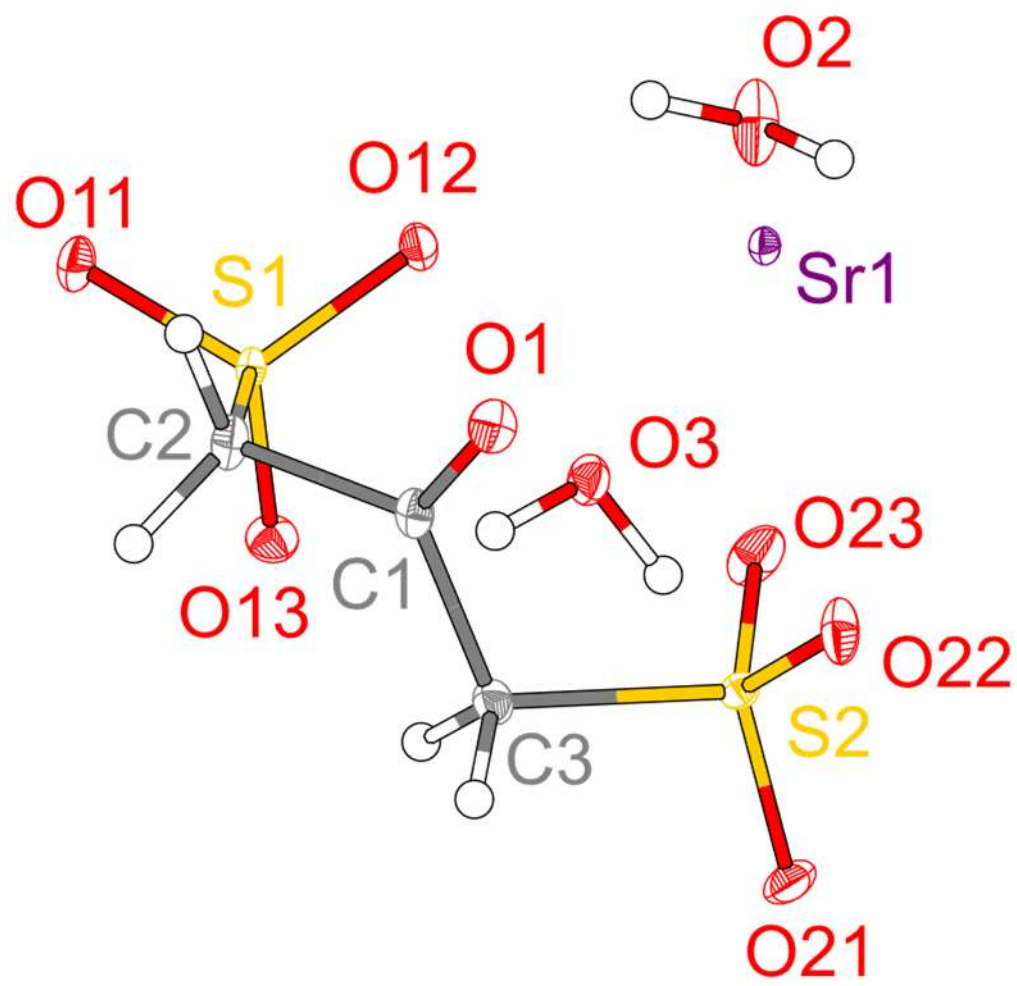

Figure S8: Thermal ellipsoid plot of the asymmetric unit of $Sr[(O_3SCH_2)_2CO](H_2O)_2$. Thermal ellipsoids shown with 50% probability.

Table S37: Fractional Atomic Coordinates (×10[4]) and Equivalent Isotropic Displacement Parameters (Å^2×10^3) for $Sr[(O_3SCH_2)_2CO](H_2O)_2$. U_{eq} is defined as 1/3 of the trace of the orthogonalised U_{IJ} tensor.

Atom	*x*	*y*	*z*	U(eq)
Sr1	3387.5(2)	6069.4(3)	7446.8(2)	6.40(7)
S1	3393.8(6)	11021.8(8)	6324.7(5)	6.90(13)
S2	4293.8(6)	6234.2(8)	4336.8(6)	7.26(12)
O1	1891.3(19)	8570(3)	4100.5(17)	12.3(4)
O2	1374(2)	6072(3)	6334(2)	20.4(5)
O3	5420.4(16)	7829(2)	7554(2)	12.5(4)
O11	3007.1(18)	12726(3)	6856.2(17)	12.4(4)
O12	2819.4(17)	9397(3)	6804.1(17)	10.6(4)
O13	4727.0(18)	10847(3)	6230.5(17)	12.4(4)
O21	5514.1(19)	5764(3)	3931.0(19)	14.8(4)
O22	3316.2(18)	5218(3)	3792.5(18)	14.6(4)
O23	4234(2)	6111(3)	5520.6(18)	18.0(5)
C1	2840(2)	9359(3)	4353(2)	9.0(4)
C2	2793(3)	11170(3)	4965(3)	11.2(5)
C3	4089(3)	8613(4)	4005(2)	10.2(5)

Table S38: Anisotropic Displacement Parameters (Å^2×10^3) for $Sr[(O_3SCH_2)_2CO](H_2O)_2$. The anisotropic displacement factor exponent takes the form: $-2\pi^2[h^2a^{*2}U_{11}+2hka^*b^*U_{12}+\ldots]$.

Atom	U_{11}	U_{22}	U_{33}	U_{23}	U_{13}	U_{12}
Sr1	7.62(11)	5.07(10)	6.50(11)	-0.37(12)	-0.28(11)	-0.27(7)
S1	8.1(3)	4.6(3)	8.0(3)	-0.6(2)	-0.8(2)	-0.05(19)
S2	7.2(3)	8.0(3)	6.6(3)	-0.4(2)	-0.2(2)	0.91(19)
O1	12.5(9)	12.2(9)	12.2(10)	-1.3(7)	-2.6(7)	-0.3(7)
O2	21.7(11)	10.7(11)	28.7(14)	-0.2(9)	-14.8(10)	0.0(8)
O3	10.4(8)	9.1(8)	18.1(10)	0.9(9)	-0.3(8)	1.5(6)
O11	16.3(9)	7.3(9)	13.5(9)	-2.8(7)	-0.8(8)	0.1(7)
O12	11.2(9)	7.4(8)	13.2(9)	1.2(7)	0.4(7)	-0.5(7)
O13	8.7(9)	14.2(9)	14.4(10)	0.5(7)	-0.9(7)	-0.2(7)
O21	9.8(9)	13.9(9)	20.8(10)	-6.3(8)	4.3(8)	1.2(7)
O22	13.1(10)	7.3(9)	23.5(12)	-0.3(8)	-6.4(8)	-1.0(7)
O23	28.2(12)	17.5(11)	8.3(10)	1.1(7)	2.0(9)	9.3(8)

C1	12.8(13)	7.7(10)	6.5(10)	1.9(9)	-2.6(10)	1.4(9)
C2	15.5(16)	7.1(11)	11.2(11)	-0.4(9)	-2.4(13)	1.6(8)
C3	11.4(12)	8.5(11)	10.9(12)	-0.6(9)	0.6(9)	-1.9(9)

Table S39: Bond Lengths and Interatomic Distances [in pm] for $Sr[(O_3SCH_2)_2CO](H_2O)_2$.

Atom	Atom	Length/pm	Atom	Atom	Length/pm
Sr1	O1[1]	273.3(2)	S1	O13	146.0(2)
Sr1	O2	257.8(2)	S1	C2	179.0(3)
Sr1	O3	255.64(18)	S2	O21	145.7(2)
Sr1	O11[2]	256.4(2)	S2	O22	145.5(2)
Sr1	O12	261.2(2)	S2	O23	145.2(2)
Sr1	O21[3]	254.7(2)	S2	C3	178.6(3)
Sr1	O22[1]	255.4(2)	O1	C1	122.0(3)
Sr1	O23	252.9(2)	C1	C2	151.2(4)
S1	O11	145.9(2)	C1	C3	152.3(4)
S1	O12	145.7(2)			

[1]1/2-X,+Y,1/2+Z; [2]+X,-1+Y,+Z; [3]1-X,1-Y,1/2+Z.

Table S40: (Reduced) Bond Angles for $Sr[(O_3SCH_2)_2CO](H_2O)_2$. Angles including Strontium atoms were omitted for clarity.

Atom	Atom	Atom	Angle/°	Atom	Atom	Atom	Angle/°
O23	/1	O21[3]	119.93(7)	O22	S2	C3	107.1(1)
O23	/1	O22[1]	150.11(8)	O23	S2	O21	111.5(1)
O11	S1	O13	113.3(1)	O23	S2	O22	113.1(1)
O11	S1	C2	104.9(1)	O23	S2	C3	106.3(1)
O12	S1	O11	112.5(1)	O1	C1	C2	120.3(2)
O12	S1	O13	112.8(1)	O1	C1	C3	121.1(2)
O12	S1	C2	105.4(1)	C2	C1	C3	118.5(2)
O13	S1	C2	107.1(1)	C1	C2	S1	113.3(2)
O21	S2	C3	105.2(1)	C1	C3	S2	113.0(2)
O22	S2	O21	113.0(1)				

[1]1/2-X,+Y,1/2+Z; [2]+X,-1+Y,+Z; [3]1-X,1-Y,1/2+Z; [4]1/2-X,+Y,-1/2+Z; [5]+X,1+Y,+Z; [6]1-X,1-Y,-1/2+Z.

Table S41: (Reduced) Torsion Angles for $Sr[(O_3SCH_2)_2CO](H_2O)_2$. Angles including Strontium atoms were omitted for clarity.

A	B	C	D	Angle/°	A	B	C	D	Angle/°
O1	C1	C2	S1	113.5(2)	O21	S2	C3	C1	179.1(2)
O1	C1	C3	S2	-51.3(3)	O22	S2	C3	C1	58.5(2)
O11	S1	C2	C1	-166.9(2)	O23	S2	C3	C1	-62.6(2)
O12	S1	C2	C1	-47.9(2)	C2	C1	C3	S2	132.3(2)
O13	S1	C2	C1	72.5(2)	C3	C1	C2	S1	-70.0(3)

[1]1/2-X,+Y,-1/2+Z; [2]+X,1+Y,+Z; [3]1-X,1-Y,-1/2+Z.

Table S42: Hydrogen Atom Coordinates (Å×10^4) and Isotropic Displacement Parameters (Å^2×10^3) for $Sr[(O_3SCH_2)_2CO](H_2O)_2$.

**Atom**	***x***	***y***	***z***	**U(eq)**
H2A	1005.7	7079.59	6130.7	31
H2B	1216.6	5286.88	5814.56	31
H3A	6022.36	7113.35	7344.59	19
H3B	5419.51	8726.56	7077.29	19
H2C	1929.04	11595.1	5000.44	13
H2D	3267.94	12103.99	4551.95	13
H3C	4742.43	9341.76	4367.2	12
H3D	4182.45	8773.26	3205.44	12

$Ca[(O_3SCH_2)_2CO](H_2O)_2$ (7)

Table S43: Crystallographic data of Bond $Ca[(O_3SCH_2)_2CO](H_2O)_2$.

Empirical formula	$C_3H_8CaO_9S_2$
Formula weight	292.29 g/mol
Temperature	100.00 K
Crystal system	Monoclinic
Space group	*C*2/*c* (No. 15)
Unit cell dimensions	a = 762.02(8) pm<br>b = 804.48(9) pm<br>c = 1613.1(2) pm<br>α = 90°<br>β = 100.540(4)°<br>γ = 90°
Volume	972.2(2) $Å^3$
Z	4
ρ_{calc}	1.997 g/cm^3
μ	1.105 mm^{-1}
F(000)	600
Radiation	MoK_α (λ = 0.71073 nm)
Crystal size	0.07 x 0.04 x 0.03 mm^3
2Θ range for data collection	5.138 to 60.994
Index ranges	$-10 \leq h \leq 10$. $-10 \leq k \leq 11$, $-22 \leq l \leq 23$
Reflections collected	6803
Independent reflections	1458 [R_{int} = 0.0242, R_σ = 0.0198]
Completeness	99.1%
Absorption correction	multiscan
Min. and max. transmission	0.649 / 0.747
Data/restraints/parameters	1458/0/87
Goodness-of-fit on F^2	1.152
Final *R* indexes [I≥2σ (I)]	R_1 = 0.0451, wR_2 = 0.0846
Final *R* indexes [all data]	R_1 = 0.0564, wR_2 = 0.0918
Largest diff. peak/hole	0.48/–0.60 e · $Å^{-3}$
CCDC-No.	2544634

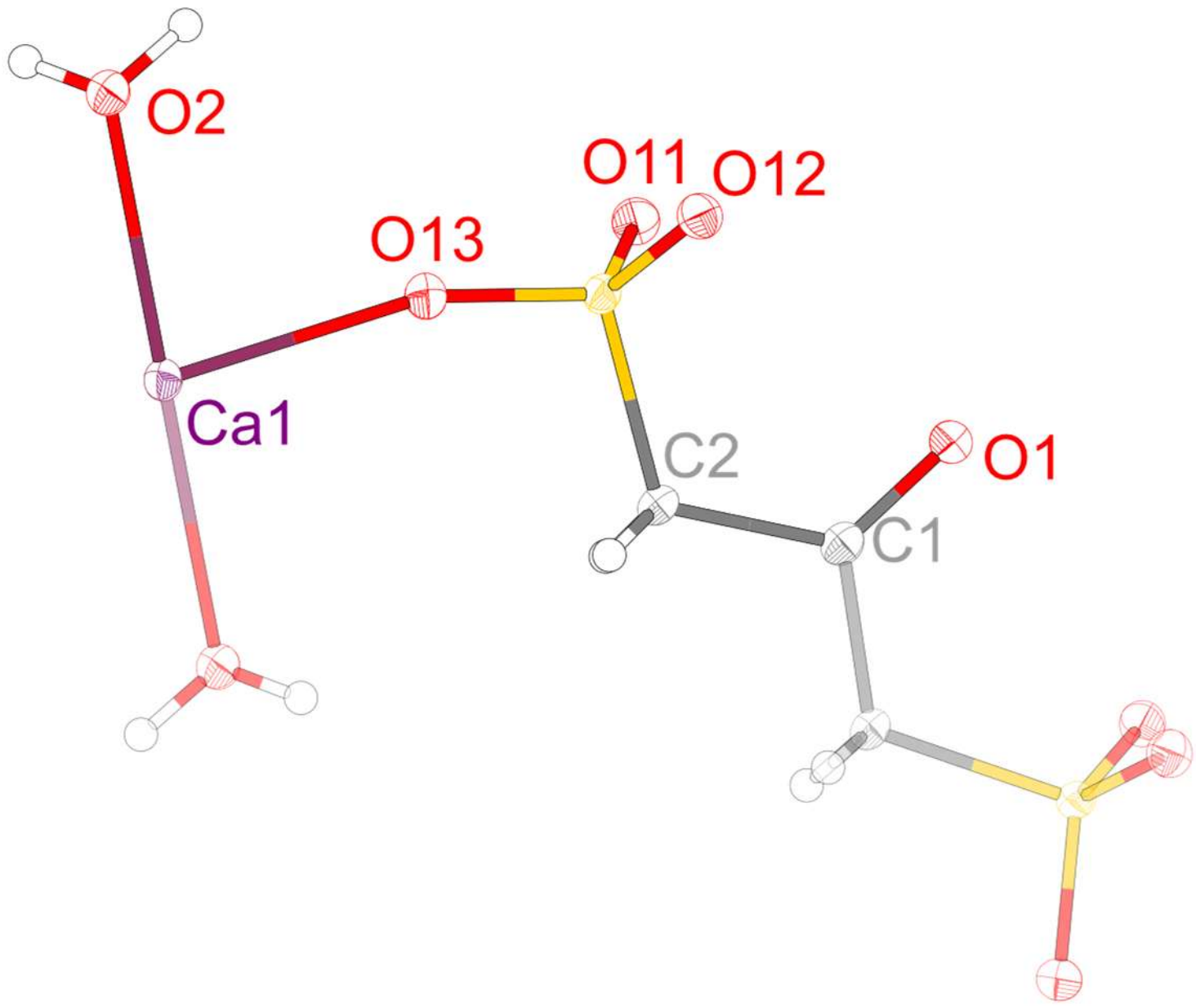

Figure S9: Thermal ellipsoid plot of the asymmetric unit of $Ca[(O_3SCH_2)_2CO](H_2O)_2$. Thermal ellipsoids shown with 50% probability. Atoms generated by symmetry operations are shown at 50% visibility.

Table S44: Fractional Atomic Coordinates ($\times10^4$) and Equivalent Isotropic Displacement Parameters ($Å^2\times10^3$) for $Ca[(O_3SCH_2)_2CO](H_2O)_2$. U_{eq} is defined as 1/3 of the trace of the orthogonalised U_{IJ} tensor.

Atom	*x*	*y*	*z*	U(eq)
Ca1	2500	2500	5000	11.70(16)
S1	3176.8(8)	6370.1(8)	3857.5(4)	11.25(14)
O1	5000	7914(4)	2500	17.8(6)
O2	3455(3)	963(3)	3907.8(14)	16.2(4)
O11	1592(2)	7247(3)	3449.5(13)	15.0(4)
O12	4540(3)	7503(3)	4297.5(12)	14.4(4)
O13	2789(3)	5035(3)	4411.2(13)	15.8(4)
C1	5000	6416(5)	2500	13.2(7)
C2	4099(4)	5337(3)	3070.4(17)	13.8(5)

Table S45: Anisotropic Displacement Parameters ($Å^2\times10^3$) for $Ca[(O_3SCH_2)_2CO](H_2O)_2$. The anisotropic displacement factor exponent takes the form: $-2\pi^2[h^2a^{*2}U_{11}+2hka^*b^*U_{12}+\ldots]$.

Atom	U_{11}	U_{22}	U_{33}	U_{23}	U_{13}	U_{12}
Ca1	11.4(3)	11.2(3)	12.9(3)	0.5(3)	Ca1	11.4(3)
S1	10.9(3)	10.9(3)	12.8(3)	1.2(2)	S1	10.9(3)
O1	25.5(15)	12.0(13)	18.6(14)	0	O1	25.5(15)
O2	16.2(9)	14.0(10)	20.4(10)	-0.2(8)	O2	16.2(9)
O11	11.5(8)	15.3(9)	18.6(9)	0.9(8)	O11	11.5(8)
O12	13.7(8)	14.1(9)	15.3(9)	-1.8(7)	O12	13.7(8)
O13	18.2(9)	13.0(9)	17.5(9)	1.6(8)	O13	18.2(9)
C1	11.8(15)	14.6(17)	14.0(16)	0	C1	11.8(15)
C2	14.6(11)	13.4(12)	15.3(11)	-0.4(10)	C2	14.6(11)

Table S46: Bond Lengths and Interatomic Distances [in pm] for $Ca[(O_3SCH_2)_2CO](H_2O)_2$.

Atom	Atom	Length/pm	Atom	Atom	Length/pm
Ca1	O2	237.3(2)	S1	O12	146.4(2)

Ca1	O2[1]	237.3(2)	S1	O13	146.1(2)
Ca1	O12[2]	233.2(2)	S1	C2	176.7(3)
Ca1	O12[3]	233.2(2)	O1	C1	120.5(5)
Ca1	O13[1]	227.7(2)	C1	C2[4]	151.7(3)
Ca1	O13	227.7(2)	C1	C2	151.7(3)
S1	O11	144.8(2)			

[1]1/2-X,1/2-Y,1-Z; [2]1-X,1-Y,1-Z; [3]-1/2+X,-1/2+Y,+Z; [4]1-X,+Y,1/2-Z.

Table S47: Bond Angles for $Ca[(O_3SCH_2)_2CO](H_2O)_2$.

Atom	Atom	Atom	Angle/°	Atom	Atom	Atom	Angle/°
O2[1]	Ca1	O2	180	O13	Ca1	O13[1]	180
O12[2]	Ca1	O2[1]	92.33(7)	O11	S1	O12	112.06(12)
O12[2]	Ca1	O2	87.67(7)	O11	S1	O13	113.00(12)
O12[3]	Ca1	O2	92.33(7)	O11	S1	C2	108.08(13)
O12[3]	Ca1	O2[1]	87.67(7)	O12	S1	C2	107.53(12)
O12[2]	Ca1	O12[3]	180	O13	S1	O12	111.49(12)
O13	Ca1	O2	95.21(7)	O13	S1	C2	104.17(13)
O13[1]	Ca1	O2	84.79(7)	S1	O12	Ca1[4]	140.56(13)
O13[1]	Ca1	O2[1]	95.21(7)	S1	O13	Ca1	163.54(13)
O13	Ca1	O2[1]	84.79(7)	O1	C1	C2	124.89(16)
O13[1]	Ca1	O12[3]	92.58(7)	O1	C1	C2[5]	124.89(16)
O13	Ca1	O12[2]	92.58(7)	C2[5]	C1	C2	110.2(3)
O13[1]	Ca1	O12[2]	87.42(7)	C1	C2	S1	116.7(2)
O13	Ca1	O12[3]	87.42(7)				

[1]1/2-X,1/2-Y,1-Z; [2]1-X,1-Y,1-Z; [3]-1/2+X,-1/2+Y,+Z; [4]1/2+X,1/2+Y,+Z; [5]1-X,+Y,1/2-Z.

Table S48: Torsion Angles for $Ca[(O_3SCH_2)_2CO](H_2O)_2$.

A	B	C	D	Angle/°	A	B	C	D	Angle/°
O1	C1	C2	S1	-6.5(2)	O13	S1	O12	Ca1[1]	60.1(2)
O11	S1	O12	Ca1[1]	-172.15(16)	O13	S1	C2	C1	-167.53(17)
O11	S1	O13	Ca1	116.1(5)	C2	S1	O12	Ca1[1]	-53.5(2)
O11	S1	C2	C1	72.0(2)	C2	S1	O13	Ca1	-1.0(5)
O12	S1	O13	Ca1	-116.6(5)	C2[2]	C1	C2	S1	173.5(2)
O12	S1	C2	C1	-49.1(2)					

[1]1/2+X,1/2+Y,+Z; [2]1-X,+Y,1/2-Z.

Table S49: Hydrogen Atom Coordinates (Å×10^4) and Isotropic Displacement Parameters (Å^2×10^3) for $Ca[(O_3SCH_2)_2CO](H_2O)_2$.

Atom	*x*	*y*	*z*	U(eq)
H2A	4260(60)	1320(60)	3740(30)	38(13)
H2B	3650(60)	-90(70)	4000(30)	51(15)
H2C	4930(40)	4560(40)	3360(20)	10(8)
H2D	3190(40)	4680(40)	2730(20)	13(8)

Spectroscopic Data

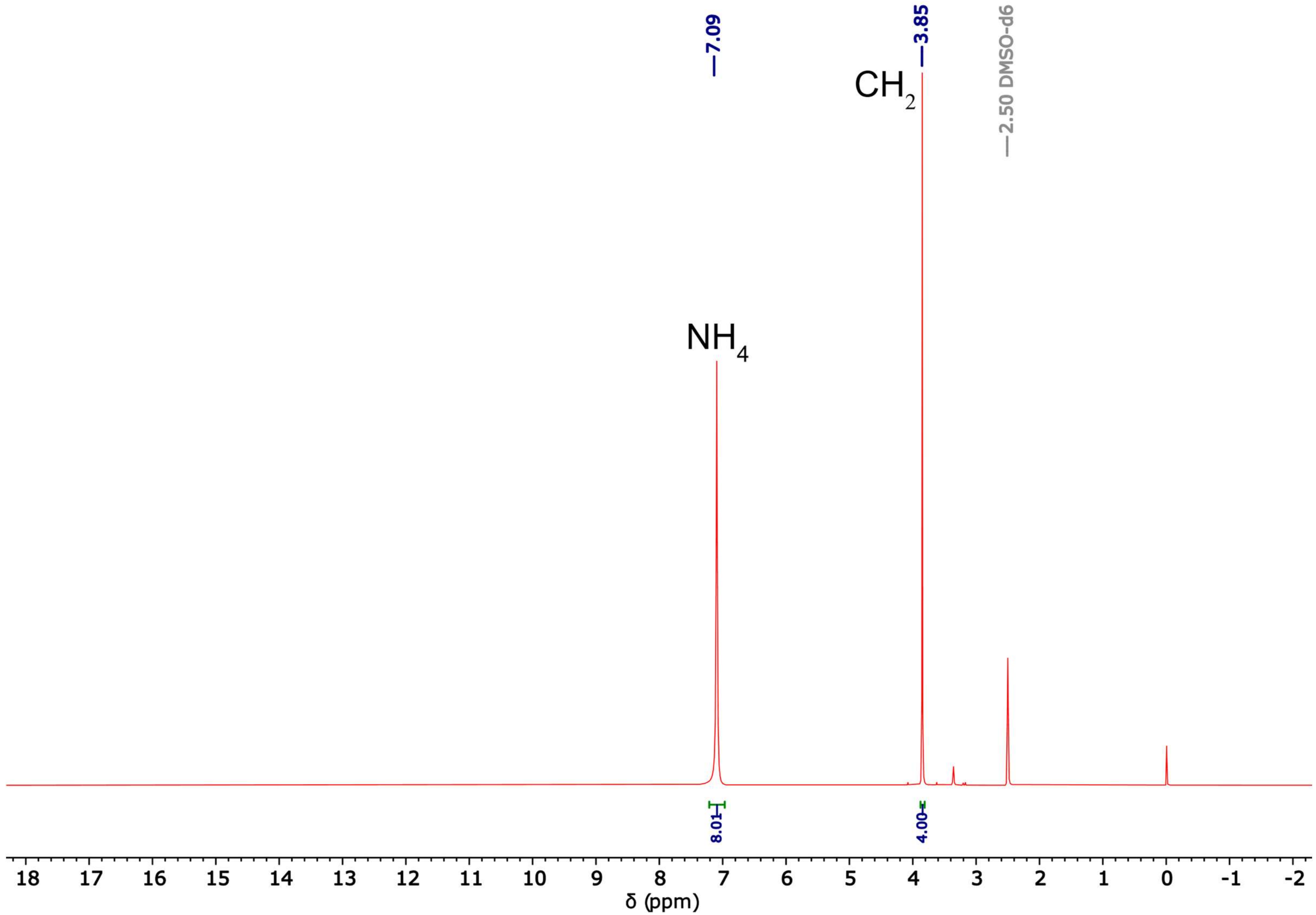

Figure S 10: ^{1}H–NMR spectrum of **2**, measured at 300 MHz and referenced on the solvent signal [DMSO–d_6].

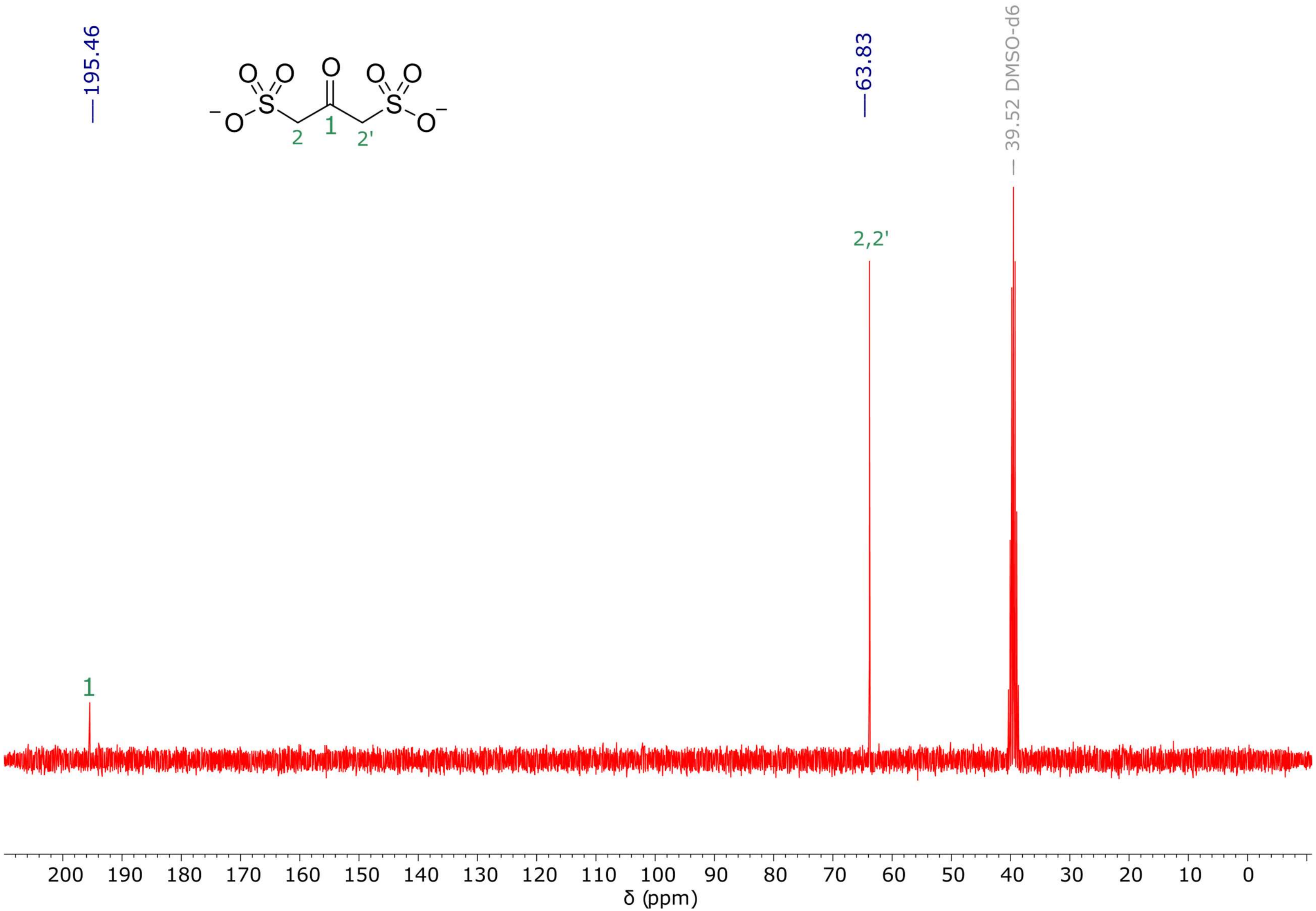

Figure S 11: ^{13}C–NMR spectrum of **2**, measured at 75 MHz and referenced on the solvent signal [DMSO–d_6].

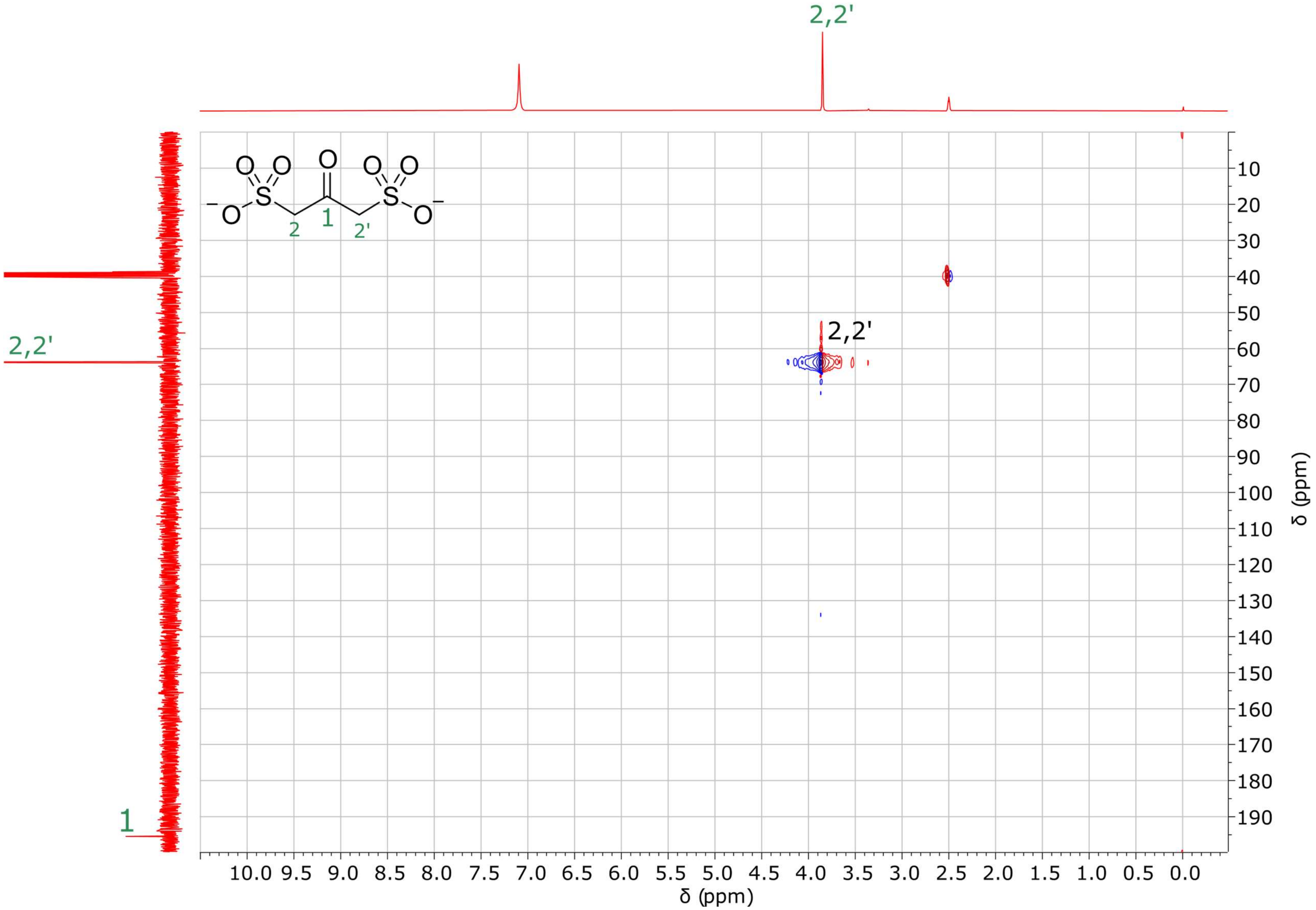

Figure S 12: $^{1}H^{13}C$–HSCQ spectrum of **2**.

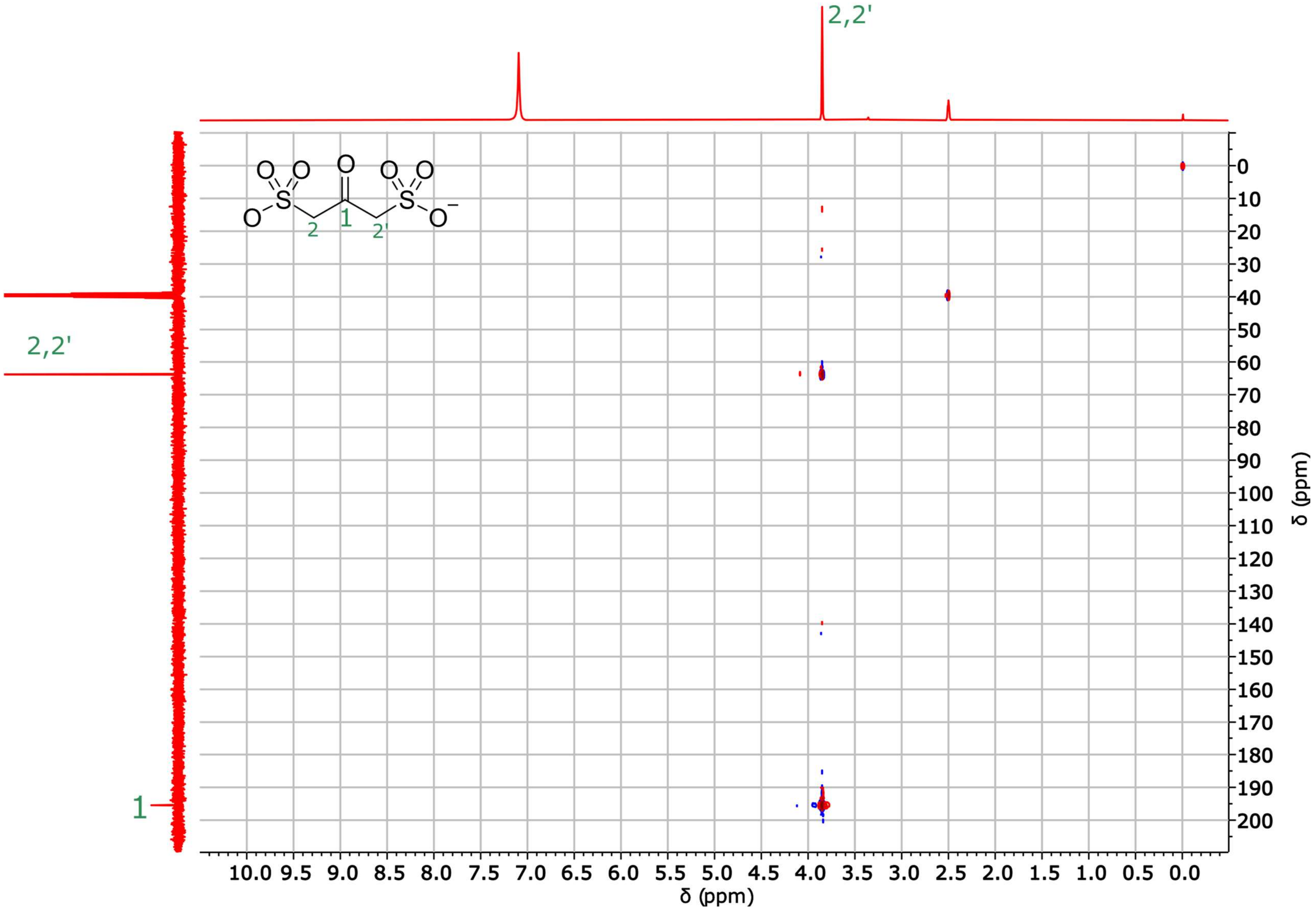

Figure S 13: $^{1}H^{13}C$–HMBC spectrum of **2**.

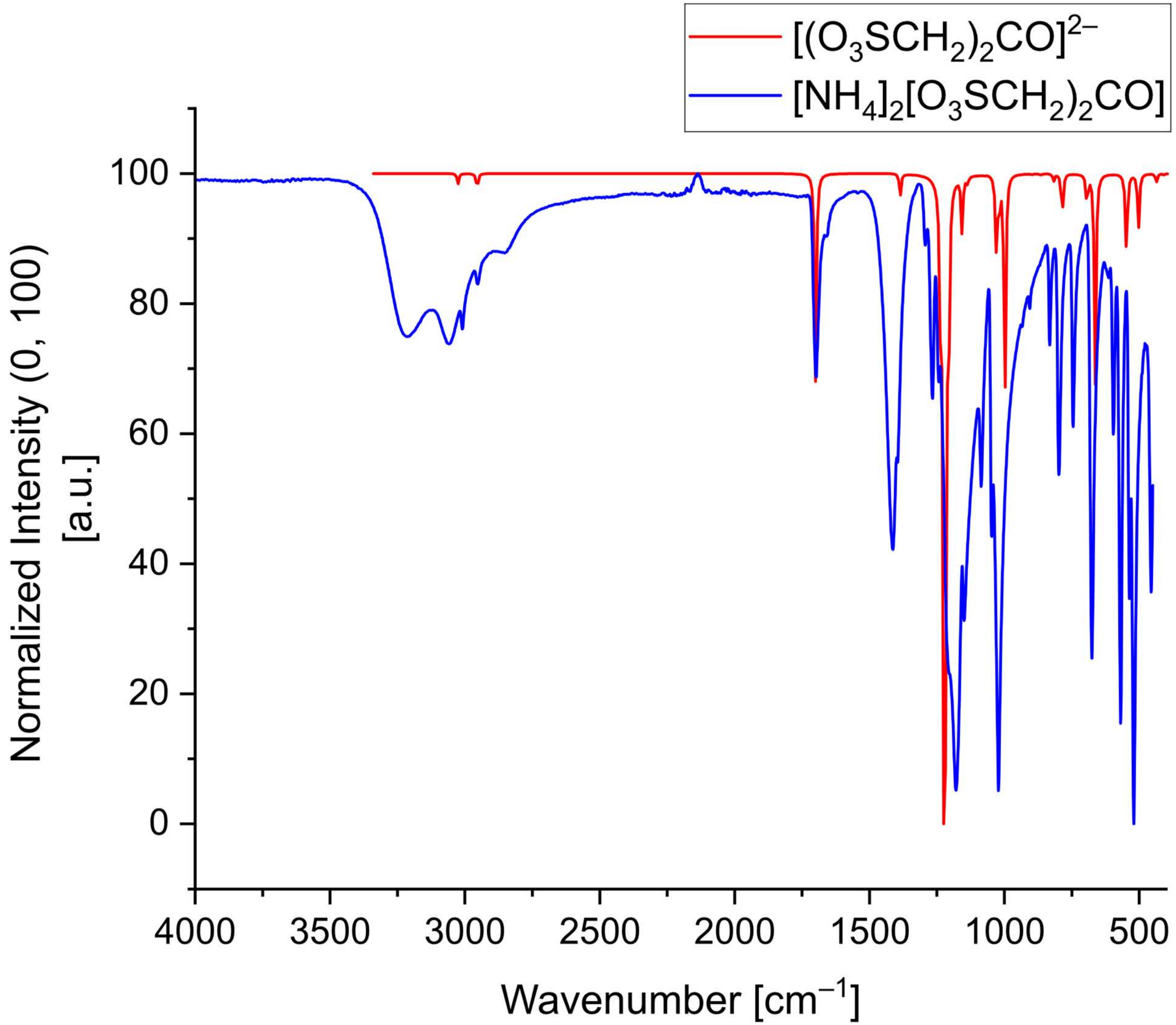

Figure S 14: FT-IR spectrum of compound **2** (blue) and the calculated IR spectrum for the $[ADS]^{2-}$ anion at the PBE0-D3/def2-TZVP level of theory (red), with an applied scale factor of 0.956. The measured intensities were normalized to [0, 100] for a better contrast using the software Origin.[2]

Table S50*:* Raman frequencies (in cm^{-1}) of compounds **1** and **2** compared to reference compounds and previously reported values.

$[NH_4][(O_3SCH_2)_2CO]$[4]	$[NH_4][(O_3SCH_2)_2CO]$ (*this work*)	$[NH_4][(O_3SCH_2)_2CO]$ (*calculated*)	Assignment
3571 – 3030	3215 (br)		ν_{as} N–H
	3064 (br)		
	3009	3025	ν_{as} C–H
	2949	2951	
1724 (br)	1699	1700	ν_s C=O
1449 (w)	1413	1385	
1351 (w)	1266	1225	ν_{as}
1333	1179		
1250 – 1163	1149	1158	δ_s C–H
	1085		
1124	1047	1031	ν_s S–O / δ_s C–C
1036	1022	997	
1010	831	816	ν_{as} C–C
971	799	783	
935	746	696	
840	675	663	
833	596		Lattice vibrations
781	569	549	
769	536		
664	520	502	
	456		ν_s C–S
		435	

as = asymmetric, s = symmetric, def. = deformation. br = broad, w = weak

Unsucessful Reactions

Table S 51: Summary of the unsuccessful reactions of 1 and 2 with different salts. The respective products were identified by single crystal X-Ray diffraction.

Reagent A	*Reagent B*	*Solvent*	*Temperature*	*Product*
ADSA	Li_2CO_3	H_2O	25 °C	Li_2CO_3
ADSA	LiBr	H_2O	25 °C	$LiHSO_4$
ADSA	CsBr	H_2O	25 °C	$CsHSO_4$
ADSA	CsOH	H_2O	25 °C	$CsHCO_3$
ADSA	Ca(OH)2	H_2O	25 °C	$CaSO_4(H_2O)_2$
$[NH_4]_2$ADS	$MgCO_3$/$Mg(OH)_2$	H_2O	25 °C	$[NH_4]_2$ADS
$[NH_4]_2$ADS	$Ba(OH)_2{\cdot}8H_2O$,	H_2O	25 °C	$[NH_4]_2$ADS
$[NH_4]_2$ADS	$CsNO_3$	H_2O	25 °C	$CsNO_3$
$[NH_4]_2$ADS	$AgNO_3$	H_2O	25 °C	$AgNO_3$

References

[1] Bruker Optik GmbH, *OPUS 6.5* **2009**, Deutschland.
[2] OriginLab Corporation, *OriginPro, Version 2024* **2024**, Northampton, MA, USA.
[3] MestrelabResearch, *MestReNova V. 15.0.1* **2024**.
[4] W. Grot, *J. Org. Chem.* **1965**, *30*, 515-517.
